# Differentiation in the *Trochulus hispidus* complex and related taxa (Pulmonata: Hygromiidae): morphology, ecology and their relation to phylogeography

**DOI:** 10.1093/mollus/eyu023

**Published:** 2014-05-14

**Authors:** Michael Duda, Luise Kruckenhauser, Helmut Sattmann, Josef Harl, Katharina Jaksch, Elisabeth Haring

**Affiliations:** 1 3rd Zoological Department, Museum of Natural History Vienna, Burgring 7, 1010 Vienna, Austria; 2 Central Research Laboratories, Museum of Natural History Vienna, Burgring 7, 1010 Vienna, Austria; 3 Department of Integrative Zoology, University of Vienna, Althanstraße 14, 1090 Vienna, Austria

## Abstract

In this study we investigated the morphology and ecology of representatives of the taxonomically ambiguous genus *Trochulus*. The main focus was on the *T. hispidus* complex, which comprises several genetically highly divergent mitochondrial clades, as determined in a parallel molecular genetic study. We analysed shell morphology and anatomical traits and asked whether the clades are differentiated in these characters. In addition, the related species *T. oreinos* and *T. striolatus* were investigated and compared with the *T. hispidus* complex. Finally, we compared the ecological requirements of the taxa. Among the genetic clades of the *T. hispidus* complex there was no clear morphological differentiation and geographic populations could not be distinguished based on their morphology. The investigated characters of the genital anatomy did not allow discrimination of any of the *T. hispidus* clades and were not even diagnostic for the group as a whole. The morphotype of *T. sericeus* is present in all clades and thus cannot be assigned to a genetic group or any specific population. Thus, our morphological data do not provide evidence that any of the mitochondrial *T. hispidus* clades represent separate species. Concerning interspecific delimitation, the *T. hispidus* complex was clearly differentiated from *T. striolatus* and *T. oreinos* by shell morphological and anatomical characters, e.g. sculpture of shell surface and details of the penis. Finally, the habitat of *T. oreinos* is different from those of the other two species. In contrast to the lack of correspondence between genetic and morphological differentiation within the *T. hispidus* complex, related species display intraspecific morphological differentiation corresponding with mitochondrial clades: within *T. striolatus* there was a slight morphological differentiation between the subspecies *T. s. striolatus*, *T. s. juvavensis* and *T. s. danubialis*. The two subspecies of *T. oreinos* could be discriminated by a small but consistent difference in the cross-section of the penis. The unequal levels of intraspecific differentiation are caused by different evolutionary histories as a consequence of disparities in ecological demands, dispersal ability and use of glacial refugia: both the *T. hispidus* complex and *T. striolatus* are fast-spreading, euryoecious organisms which are able to (re-)colonize habitats and survive under different climate conditions. While the *T. hispidus* complex probably survived the Pleistocene in several glacial refugia, for *T. striolatus* one glacial refugium is suggested. *Trochulus oreinos* differs from the other taxa, as it is a slow disperser with a narrow ecological niche. We suggest that its subspecies spent at least the last glaciation in or close to the presently inhabited areas.

## INTRODUCTION

The classification of species and subspecies in Central European terrestrial gastropods is still disputed in many cases. One reason is that reliable morphological characters differentiating the taxa are scarce. Moreover, varying species concepts have led to contradictory taxonomic classifications, which in some cases have also been influenced by conservation aspects. Some authors (e.g. Falkner, 1991; Reischütz, 1999) introduced ‘moderate splitting’ by describing slightly deviating morphological forms as subspecies. This is potentially useful as an argument to protect local populations threatened by habitat destruction. The introduction of molecular genetic methods in biological systematics has often contributed to solving taxonomic problems. This approach has, however, frequently caused even more confusion by revealing more complex patterns of hitherto unnoticed genetic variation and differentiation of mitochondrial (mt) clades (Sauer & Hausdorf, 2012).

One example is the genus *Trochulus* Chemnitz, 1786. This genus has frequently been the focus of taxonomic questions, which have been addressed using morphological (Focart, 1965; Gittenberger, Backhuys & Ripken, 1970; Schileyko, 1978; Falkner, 1995; Falkner, Ripken & Falkner, 2002; Proćków, 2009; Duda *et al*., 2011) and genetic data (Pfenninger *et al*., 2005; Dépraz, Hausser & Pfenninger, 2009; Kruckenhauser *et al*., 2014). The species with the widest distribution within the genus is *T. hispidus* (Linnaeus, 1758). It prefers moist habitats from the northern parts of the Mediterranean peninsulas (Iberian, Apennine and Balkan) northwards to Scandinavia and eastwards to the Urals (Ložek, 1956). Reports from Sardinia were likely based on confusion with *Ichnusotricha berninii* (Giusti & Manganelli, 1987). Based on its high shell variability, several attempts have been made to divide *T. hispidus* into different species or subspecies (Focart, 1965; Schileyko, 1978). These, however, have been criticised and are not commonly accepted (Gittenberger *et al*., 1970; Naggs, 1985; Proćków, 2009). Additionally, some conchologically similar species, particularly *T. plebeius*, *T. sericeus* and *T. coelomphala*, have been considered as valid species by some authors (e.g. Falkner, Bank & von Proschwitz, 2000), while other authors have suggested merging at least some of them with *T. hispidus* (e.g. Proćków, 2009). Based on molecular analyses, some authors have suggested splitting *T. hispidus* into several cryptic species (Pfenninger *et al*., 2005; Dépraz *et al*., 2009). In a survey of *Trochulus* species from Germany, Switzerland and France, Pfenninger *et al.* (2005) found several highly distinct mt clades which could, however, not be classified unambiguously. Due to the complicated taxonomic situation and the ambiguous differentiation of *T. hispidus* and *T. sericeus*, Dépraz *et al.* (2009) suggested that these taxa should be subsumed under the term ‘*T. hispidus/sericeus* complex’. We have subsumed such snails appearing in the various mt clades detected by Kruckenhauser *et al*. (2014) under the more general term ‘*T. hispidus* complex’ to account for the high mt variation of snails with a *T. hispidus-*like morphology.

Beside *T. hispidus*, several related species occur in Austria and the surrounding countries, among them *T. oreinos* (A. J. Wagner, 1915), *T. striolatus* (C. Pfeiffer, 1828), *T. coelomphala* (Loccard, 1888), *T. clandestinus* (Hartmann, 1821), *T. villosus* (Draparnaud, 1805), *T. villosulus* (Roßmässler, 1838) and *T. biconicus* (Eder, 1917).

**Figure 1.**
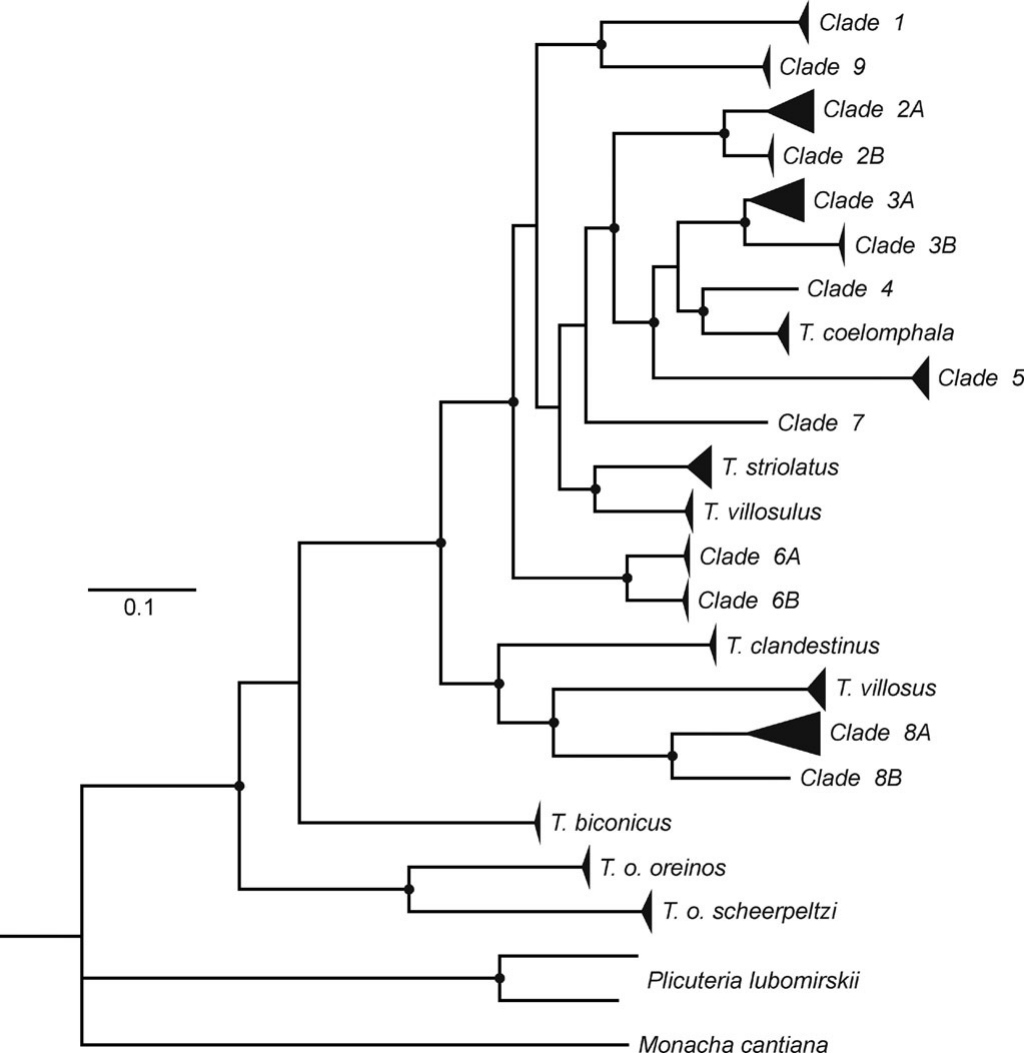
Schematic tree based on partial sequences of COI, 16S rRNA and 12S rRNA genes of *Trochulus* species and related taxa. Clades 1–9: different mitochondrial clades of the *T. hispidus* complex (modified after Kruckenhauser *et al*., 2014).

In a genetic analysis comprising mainly Austrian populations of the *T. hispidus* complex as well as other species, we revealed a large group of *Trochulus* (Kruckenhauser *et al*., 2014) containing 16 mt clades separated by remarkably high distances (Fig. 1). Two of them, representing the species *T. biconicus* and *T. oreinos*, were clearly separated in the tree. Another five of the clades represented morphologically more or less well-defined species, which were interspersed among nine clades containing individuals of ‘typical’ *T. hispidus* appearance (flattened shell with wide umbilicus), as well as specimens with a more globular shell and narrow umbilicus. The latter appearance tentatively conforms to descriptions of the problematic taxon *T. sericeus*. Yet, for many individuals such an assignment to *T. sericeus* proved to be not feasible, as the characters varied widely. Moreover, *T. hispidus* is paraphyletic according to the mt tree and an assignment of the taxa to specific clades remained ambiguous.

These complicated relationships raise questions about the status of the species *T. hispidus* and whether the clades of the *T. hispidus* complex—or at least some of them—might represent distinct species. To address this question, the central aim of the present study was to determine whether snails belonging to distinct mt clades were distinguishable by morphometric traits not visible by cursory inspection. The large sample of genetically determined individuals from Austria and surrounding countries permitted a comprehensive morphological investigation including the same individuals. We connected our results with analyses of habitat preferences.

Two of the related species investigated by Kruckenhauser *et al.* (2014), *T. oreinos* and *T. striolatus*, were available in sufficient numbers to be included in the morphological and ecological analyses. *Trochulus oreinos*, an Austrian endemic from the northern calcareous Alps (Klemm, 1974), is characterized by a small flat shell and tiny curved hairs. It was originally considered to be a local subspecies of *T. hispidus* (Wagner, 1915), but was later split as a separate species (Falkner, 1982, 1995). The latter view was confirmed by genetic and morphological data (Duda *et al*., 2011; Kruckenhauser *et al*., 2014) as well as ecological data (Duda *et al*., 2010). *Trochulus oreinos* comprises two geographically separated subspecies, *T. o. oreinos* (Wagner, 1915) and *T. o. scheerpeltzi* (Mikula, 1954), which overlap in shell morphology but are genetically distinct (for details see Duda *et al*., 2011 and Kruckenhauser *et al*., 2014).

*Trochulus striolatus* has the second-widest distribution within the genus. It occurs from Ireland and Great Britain across France and Germany to Austria and along the River Danube in southern Slovakia and northern Hungary (Kerney, Cameron & Jungbluth, 1983; Proćków, 2009). Its shell was described as larger, with stronger striation and a blunt keel on the last whorl (Kerney *et al*., 1983; Falkner, 1989). According to Falkner *et al.* (2000), *T. striolatus* comprises five subspecies that have been described based on small differences in shell and genital morphology: *T. s. striolatus* (Pfeiffer, 1828) in western Germany and northern Switzerland, *T. s. danubialis* (Clessin, 1874) along the River Danube from Bavaria to Hungary, *T. s. juvavensis* (Geyer, 1914) restricted to a few mountains in the northeastern calcareous Alps, *T. s. austriacus* (Mahler, 1952) in the northeastern Alps and *T. s. abludens* (Locard, 1888) in The Netherlands, France, Great Britain and Ireland.

**Figure 2.**
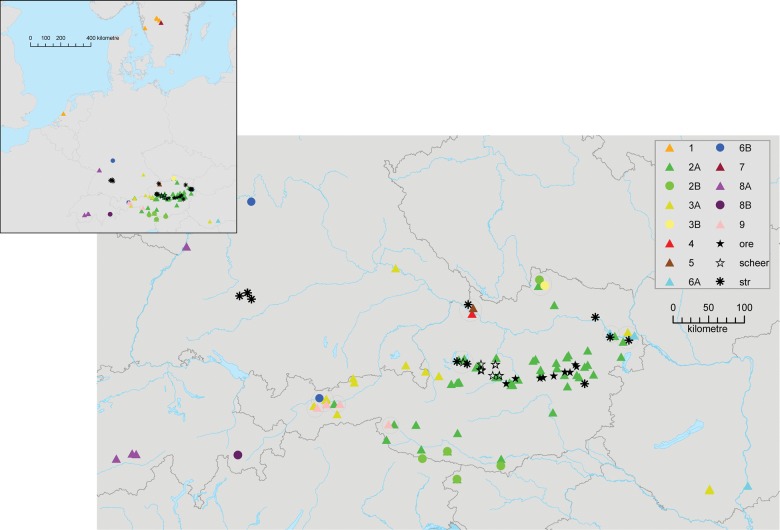
Distribution of investigated clades and taxa of *Trochulus* (modified after Kruckenhauser *et al*., 2014) in Europe and Austria. 1–9 are different mitochondrial clades of the *T. hispidus* complex. Abbreviations: ore, *T. o. oreinos*; scheer, *T. o. scheerpeltzi*; str, *T. striolatus* subspecies.

The morphological and anatomical investigations presented here include populations representing the *T. hispidus* complex as well as *T. oreinos* and *T. striolatus* (for sample localities see Fig. 2)*.* The following questions were addressed: (1) Are the clades of the *T. hispidus* complex differentiated with respect to shell morphology? (2) Is there any morphologically differentiated group corresponding to any of the clades detected within the *T. hispidus* complex by Kruckenhauser *et al*. (2014) that can be ascribed to *T. sericeus*? (3) Is there any difference in the genital anatomy that characterizes, or separates, *T. hispidus* from *T. sericeus*? We searched for qualitative traits that are characteristic for one or several certain clades. (4) Are there morphological and anatomical characters clearly differentiating *T. hispidus* from the related species *T. striolatus* and *T. oreinos*? In a final step, we discuss habitats of the various taxa (*T. hispidus* complex, *T. oreinos* and *T. striolatus*) to consider the differentiation of mt clades with respect to ecological and biogeographic factors.

Overall, these analyses explore the general possibilities and limitations of classical morphological analyses in snails. Furthermore, the combined genetic and morphological results should help to clarify unresolved systematic issues. We also discuss conservation aspects of populations belonging to different mt clades of the *T. hispidus* complex in connection with landscape development.

## MATERIAL AND METHODS

### Specimens, data sampling and documentation

The number of investigated specimens was predetermined by the genetic study of Kruckenhauser *et al.* (2014). From that dataset, 253 individuals, which appeared to be adult or close to maturity (as defined by Duda *et al*., 2011), were selected (details including GenBank accession numbers were listed by Kruckenhauser *et al*., 2014). The total number of sample sites was 108. At two sites (86, 93) only genetic data and habitat parameters were documented as there were no adult individuals of *Trochulus*. Numbers of specimens from each site and for each methodological approach are summarized in Tables 1 and 2. The samples analysed in this study also included those individuals that had been analysed both morphologically and genetically by Duda *et al*. (2011). For maximum comparability with the genetic study we included individuals of all clades, even if the numbers were small. Consequently, some clades could not be included in all analyses. However, the measurements are provided for all individuals (except subadult individuals of clades 4 and 7). Figure 2 shows a geographic overview of sample sites, clades and species. Raw data of measurements and the documentation of the habitats are summarized in the Supplementary Material (Tables S1 and S2).

**Table 1.** Sample sites of the *Trochulus hispidus* complex.

Country	Locality	SNr	Alt	Clade	H	G	M	A
The Netherlands	Leiden, Valkenburgske Meer	418	−23	1	1	3	3	3
Sweden	Västra Götalands Iän, Kvänum	451	85	1	0	2	2	1
Sweden	Göteborg, Botanical garden	452	15	1	0	2	1	1
Sweden	Västra Götalands Iän, Falköping	454	217	1	0	1	1	1
Sweden	Västra Götalands Iän, Norra Vånga	455	110	1	0	2	2	2
Austria	**Donauauen, Orth, Altarm**	3	145	2A	0	4	4	0
Austria	Semmering, Maria Schutz	5	871	2A	1	3	2	0
Austria	Johnsbachtal, Langriesmündung	24	652	2A	1	3	2	0
Austria	Johnsbachtal, Kneippstation	32	865	2A	1	3	2	0
Austria	**Donauauen, Regelsbrunner Arm**	33	147	2A	1	3	3	1
Austria	Hochlecken, Taferlklause	42	778	2A	0	2	1	0
Austria	Würflach, Johannesbachklamm	50	445	2A	1	3	3	0
Austria	Breitenstein, Adlitzgraben	52	650	2A	1	2	2	0
Austria	**Sattnitz, Mieger**	60	408	2A	1	3	0	0
Austria	Gailtaler Alpen, Kreuzen	64	985	2A	1	6	2	1
Austria	Gurktaler Alpen	66	950	2A	0	4	2	0
Austria	**Achensee, Achenbachtal**	93	843	2A	1	2	0	0
Austria	Hallstatt, Salzberg	102	942	2A	1	3	3	0
Austria	Dürrenstein, Lechnergraben	104	604	2A	1	3	3	0
Austria	Dürradmer, Kräuterin	130	1100	2A	1	3	3	0
Austria	Grazer Bergland, Semriach	140	503	2A	1	10	10	1
Austria	Johnsbachtal, Kölblwirt	144	868	2A	1	3	3	0
Austria	Johnsbachtal, Wasserfallmauer	145	978	2A	1	3	3	0
Austria	Hallstatt, Waldbachstrub	157	806	2A	0	4	4	0
Austria	Hallstatt, Sportplatz	158	524	2A	1	3	2	0
Austria	**Gmünd, Kurzschwarza**	159	551	2A	1	8	8	2
Austria	Hallstatt, Klausalm	160	796	2A	1	3	2	0
Austria	Pittental, Schlattenbach	167	397	2A	1	3	2	0
Austria	Sierningtal, Stixenstein	168	470	2A	1	3	3	0
Austria	Innervillgraten, Kalkstein	204	1620	2A	1	4	3	0
Austria	Gailtaler Alpen, Laas	205	920	2A	0	3	3	0
Austria	**Defereggen Gebirge, Obermauern**	207	1320	2A	0	1	0	0
Austria	Fischbacher Alpen, Hauereck	208	1187	2A	0	2	1	0
Austria	Seewaldtal, Bach	215	1090	2A	1	1	1	0
Slovenia	**Soča valley, Soča**	223	435	2A	1	2	2	0
Austria	Donauinsel, Neue Donau	231	165	2A	1	3	2	0
Austria	Warscheneck, Wurzeralmbahn	237	810	2A	1	1	1	1
Austria	Salzkammergut, Hochalm	285	663	2A	0	1	1	1
Austria	Neusiedler See, West shore	286	124	2A	0	2	2	0
Austria	Frein, Freinbach	306	869	2A	0	3	3	0
Austria	Göller, Gscheid	311	914	2A	1	3	3	0
Austria	Tiefental, Ochbauer	313	739	2A	1	3	3	1
Austria	Berndorf, Grabenweg	315	412	2A	1	3	3	0
Austria	Halbachtal, Rossbachklamm	317	649	2A	1	3	3	1
Austria	Salzatal, Weichselboden	318	660	2A	1	3	2	0
Austria	Großer Phyrgas, Arlingsattel	319	1425	2A	1	2	1	0
Austria	Johnsbachtal, Kölblalm	323	1076	2A	1	2	2	0
Austria	Hieflau, Schneckensafari	327	523	2A	1	3	3	0
Austria	Lunz, Seehof	341	610	2A	0	2	2	0
Austria	Gosau, Talstation Zwieselbahn	361	924	2A	1	3	2	0
Austria	Almtal, Almsee	380	593	2A	1	3	3	0
Austria	Straneggbachtal, Vordere Hetzau	385	668	2A	1	3	3	0
Austria	Steyerlingtal, Schattseite	386	485	2A	1	2	1	0
Austria	Oberes Mölltal, Jungfernsprung	446	1148	2A	1	3	3	0
Austria	Gföhl, Neubau	534	550	2A	1	1	1	0
Austria	**Gmünd, Langschwarza**	545	552	2A	1	1	1	0
Austria	**Neu Götzens, Lufens**	548	820	2A	1	5	5	1
Austria	Gailtaler Alpen, Kreuzen	64	985	2B	1	3	2	1
Italy	Plöckenpass, Tischlbong	200	837	2B	1	3	2	2
Slovenia	**Soča valley, Soča**	223	435	2B	1	1	1	1
Austria	**Gmünd, Kurzschwarza**	159	551	2B	1	2	2	2
Austria	Hochobirmassiv, Freibach	402	733	2B	1	3	3	3
Austria	**Donauauen, Regelsbrunner Arm**	33	147	3A	1	1	1	0
Austria	Achensee, Unterautal	86	946	3A	1	1	0	0
Austria	**Achensee, Achenbachtal**	93	843	3A	1	1	0	0
Austria	Seewaldtal, Bach	215	1090	3A	1	2	2	0
Austria	Seewaldtal, Seewaldmoor	217	1048	3A	1	6	5	2
Hungary	Mecsek	288	182	3A	1	2	2	1
Hungary	Komló, Sikonda Cementry	291	195	3A	1	3	3	1
Hungary	Mánfa, Doczymalom	292	197	3A	1	3	2	1
Germany	Untersberg_Neuhäusl	407	781	3A	1	3	3	1
Germany	Ruhpolding, Mühlwinkel Brand	412	671	3A	1	3	3	1
Germany	Regensburg, Pfatter	483	160	3A	1	1	1	1
Austria	**Inntal, Hatting**	549	599	3A	1	3	3	1
Austria	**Inntal, Inzing**	550	600	3A	1	1	1	1
Austria	**Gmünd, Kurzschwarza**	159	551	3B	1	1	1	1
Austria	**Gmünd, Langschwarza**	545	552	3B	1	2	2	1
Austria	**Sauwald, Schlögen**	476	293	4	1	1	0	0
Austria	**Sauwald, Schlögen**	476	293	5	1	2	1	0
Austria	**Donauauen, Orth, Altarm**	3	145	6A	0	4	3	3
Austria	**Donauauen, Regelsbrunner Arm**	33	147	6A	1	3	3	2
Hungary	Baja, Dunafürdö	296	91	6A	1	3	3	3
Austria	**Inntal, Hatting**	549	599	6B	1	2	2	1
Germany	Wertheim, Bronnbach	482	325	6B	0	3	2	1
Sweden	Västra Götalands Iän, Yllestad	453	244	7	0	1	0	0
Switzerland	Graubünden, Sur	248	1802	8A	0	2	2	2
Switzerland	Wildhorn, Lac de Tseutsier	541	1755	8B	1	1	1	1
Germany	Eggenstein, Altrhein	555	105	8B	0	2	2	2
Germany	Eggenstein, Leopoldshafen	556	100	8B	0	2	2	2
Switzerland	Kandersteg, Lötschbergpass	561	2195	8B	0	2	2	2
Austria	**Defereggen Gebirge, Obermauern**	207	1320	9	0	1	0	0
Austria	**Neu Götzens, Lufens**	548	820	9	1	8	5	5
Austria	**Inntal, Hatting**	549	599	9	1	1	1	1
Austria	**Inntal, Inzing**	550	600	9	1	6	6	4
	Total number				69	253	212	68

Sample sites harbouring individuals of more than one mt clade (counted just once in habitat analysis) are indicated in bold. Abbreviations: SNr, sample site number; Alt, altitude (m above sea level); H, habitat analysis (0/1 = no/yes); G, number of specimens investigated genetically; M, number of specimens included in the analysis of shell morphology; A, number of specimens included in the analysis of genital anatomy.

**Table 2.** Sample sites of *Trochulus oreinos* and *T. striolatus*.

Country	Locality	SNr	Alt	Species	Subspecies	H	G	M	A
Austria	Admonter Kalbling	55	2026	*T. oreinos*	*oreinos*	1	6	6	2
Austria	Rax, Bismarcksteig	79	1787	*T. oreinos*	*oreinos*	1	6	1	1
Austria	Hochschwab, Schiestlhaus	134	2179	*T. oreinos*	*oreinos*	1	3	2	1
Austria	Hochschwab, Severinkogel	165	2010	*T. oreinos*	*oreinos*	1	1	0	0
Austria	Schneeberg, Fadenwände	172	1562	*T. oreinos*	*oreinos*	1	2	1	0
Austria	Schneeberg, Waxriegel	178	1873	*T. oreinos*	*oreinos*	1	3	3	1
Austria	Schneealpe, Schauerkogel	338	1664	*T. oreinos*	*oreinos*	1	3	3	2
Austria	Tamischbachturm	399	1940	*T. oreinos*	*oreinos*	1	3	1	1
Austria	Rax, Schlangenweg	448	1600	*T. oreinos*	*oreinos*	0	2	1	0
Austria	Hohe Veitsch	588	1979	*T. oreinos*	*oreinos*	1	3	3	2
Austria	Höllengebirge, Bledigupf	12	1677	*T. oreinos*	*scheerpeltzi*	1	1	1	1
Austria	Warscheneck, Toter Mann	132	2028	*T. oreinos*	*scheerpeltzi*	1	1	1	1
Austria	Hoher Nock, Hauptkar	351	1704	*T. oreinos*	*scheerpeltzi*	1	3	3	1
Austria	Hoher Nock, Haltersitz	367	1583	*T. oreinos*	*scheerpeltzi*	1	3	2	2
Austria	Hoher Nock, Feichtausee	369	1399	*T. oreinos*	*scheerpeltzi*	1	2	3	1
Austria	Großer Priel, Hinterer Ackergraben	382	1564	*T. oreinos*	*scheerpeltzi*	0	2	2	1
Austria	Großer Priel, Welser Hütte	383	1747	*T. oreinos*	*scheerpeltzi*	1	3	2	1
Austria	Großer Priel, Fleischbanksattel	387	2157	*T. oreinos*	*scheerpeltzi*	1	3	1	0
Austria	Großer Priel, Schlund	389	2284	*T. oreinos*	*scheerpeltzi*	1	3	2	1
Austria	Großer Phyrgas, Haller Mauern	443	1900	*T. oreinos*	*scheerpeltzi*	1	3	2	1
Austria	Großer Phyrgas, Westgrat	444	2000	*T. oreinos*	*scheerpeltzi*	1	3	2	1
				Total number	19	59	42	21	
Austria	**Donauauen, Orth, Altarm**	3	145	*T. striolatus*	*danubialis*	0	1	0	0
Austria	**Donauauen, Regelsbrunner Arm**	33	147	*T. striolatus*	*danubialis*	1	3	0	0
Austria	Wechsel, Mariensee	71	800	*T. striolatus*	*danubialis*	0	1	1	1
Austria	Stockerau, Donau Auen	142	176	*T. striolatus*	*danubialis*	1	2	1	0
Austria	Fischamend-Altarm	298	154	*T. striolatus*	*danubialis*	0	2	2	2
Austria	Sauwald-Engelhartszell	469	282	*T. striolatus*	*danubialis*	1	3	3	2
Austria	Höllengebirge, Aurach Ursprung	41	857	*T. striolatus*	*juvavensis*	0	2	0	0
Austria	**Höllengebirge, Taferlklause**	42	778	*T. striolatus*	*juvavensis*	1	1	0	0
Austria	Höllengebirge, Steinkogel	43	1531	*T. striolatus*	*juvavensis*	1	3	2	0
Austria	Pledialm, Feuerkogel	45	1444	*T. striolatus*	*juvavensis*	0	3	3	0
Austria	Hochlecken, Höllengebirge	122	1574	*T. striolatus*	*juvavensis*	1	6	3	2
Germany	Alb-Donau Kreis, Laichingen	249	750	*T. striolatus*	*striolatus*	0	2	2	0
Germany	Schwäbische Alb, Filsursprung	414	414	*T. striolatus*	*striolatus*	1	3	3	2
Germany	Schwäbische Alb, Wiesensteig	415	594	*T. striolatus*	*striolatus*	1	3	3	0
Germany	Schwäbische Alb, Grabenstetten	416	675	*T. striolatus*	*striolatus*	1	3	3	1
				Total number	9	38	26	10	

Sample sites with syntopical occurrence of *T. hispidus* complex and *T. striolatus* subspp. are indicated in bold. Abbreviations: SNr, sample site number; Alt, altitude (m above sea level); H, habitat analysis (0/1 = no/yes); G, number of specimens investigated genetically; M, number of specimens included in the analysis of shell morphology; A, number of specimens included in the analysis of genital anatomy.

Exact positions and elevations of sampling sites were determined using GPS and recorded together with habitat and landscape structures (see also Tables 3 and 4 for exact definitions). Animals were drowned in heated water as described by Kruckenhauser, Harl & Sattmann (2011) and stored in 80% ethanol. Specimens collected by colleagues were directly fixed in 96% ethanol.

**Table 3.** Definition of habitat types.

Habitat type	Definition
Open areas	
Free of vegetation (FV)	Natural or anthropogenically influenced areas with no vegetation
Meadow (ME)	Medium dry grassland, more or less intensively farmed, below subalpine ecotone
Marsh (MA)	Wet grassland vegetated by grasses, reeds and sedges, either farmed or not
High perennial herbs (HP)	Dense populations of high perennial herbs like *Urtica* and *Petasites*
Forests	
Riparian forest (RF)	Central European inundation forests along rivers, at least particularly periodically flooded
Alder carr (AC)	Forest on permanent wet locations dominated by alders (*Alnus*). No periodical flood, but consistently high soil water level
Deciduous forest (DF)	Central and northern European forests dominantly vegetated by deciduous trees, on medium moist to dry locations
Mixed forest (MF)	Central and northern European forests vegetated by deciduous and coniferous trees, on medium moist to dry locations
Coniferous forest (CF)	Central and northern European forests vegetated by coniferous trees, on medium moist to dry locations
(sub) Alpine habitats	
(sub) Alpine grassland (AG)	Natural and anthropogenically influenced meadows above lower border of subalpine ecotone on medium moist to dry places
Mountain pine shrubbery (MP)	Subalpine areas vegetated by shrubberies of mountain pines (*Pinus mugo*). Represents the highest community of closed woody vegetation in the Alps together with green alder (*Alnus viridis*) shrubbery
Habitats with strong anthropogenic interference	
Garden/park (GP)	Intensively cultivated areas dominated by lawn, ornamental plants or fruit trees, situated within or adjacent to settlement areas
Ruderal area (RA)	Areas with intensive anthropogenic disturbance but without direct cultivation or land use like construction sites or abandoned fields

**Table 4.** Definition of landscape structures.

Landscape structure	Definition
Edge of forest (EF)	Gradual or abrupt change of forest to open vegetation like meadows
Loose trees and shrubs (LT)	Expanded cover of trees and shrubs in patchy formation
Hedgerows and shrubs (HS)	Lines or small areas of shrubs which can vary in density and structure
Boundary ridge (BR)	Narrow lines of extensive green land between meadows, fields or along streets and paths
Single trees and shrubs (ST)	Single, isolated specimens of trees and shrubs
Riverbank grove (RG)	Groups or rows of trees beneath a riverbank
Single stones (SI)	Stones lying on the surface with no contact with each other
Bank/dam (BD)	Earth walls such as batteries and levees
Boulders (BO)	Stones with contact with each other, not covered by earth or vegetation
Rocks (RO)	Compact, solid *in situ* aggregation of minerals occurring naturally
Canyon/rock face (CR)	Steep, extended rock walls

For documentation all dissected animals were photographed. Shell photographs were taken with a Nikon digital sight D3-Fi1 camera fixed on different stereomicroscopes. Photos of shells and complete genital tracts were taken using a Wild M420 stereomicroscope (*T. hispidus*, *T. oreinos*) or a Leica MZ 12.5 (*T. striolatus*) at lowest magnification (5.8×, 0.8×). Penis cross sections of all taxa were examined under a Wild M420 stereomicroscope at highest magnification (35×). All photographs were created as extended depth of field images with CombineZ software (Hadley, 2010). A selection of all these photos can be found in the Supplementary Material.

### Selection of characters

For species delimitation of *Trochulus*, the selection of both shell and genital traits is problematic. Nevertheless, in some cases, combinations of these traits distinguish species by trend (Pawłowska-Banasiak, 2008; Duda *et al*., 2011). Among shell traits, especially external traits such as conspicuously distinct hair lengths and constant sculptures of shell surface allow reliable recognition in some species (Gittenberger & Neuteboom, 1991; Duda *et al*., 2011). Among anatomical traits, the basic patterns of plicae in the penis and vagina proved to be useful to differentiate species within the tribe Trochulini Lindholm, 1929 (Schileyko, 1978; Proćków, 2009), although this cannot be assumed for all Hygromiidae (see also Pawłowska-Banasiak, 2008). Conspicuous formations within the genital apparatus occurring in single species, such as the extremely prolonged inner dart sacs of *Petasina unidentata*, may provide reliable species recognition in some cases (Schileyko, 1978, 2006; Proćków, 2009). Measurements of genitalia lengths can lead to ambiguous results: they can be biased by differences within populations, by seasonal differences, retraction state of the soft body, by stretching or different positioning during measuring, or by the preservation method (Emberton, 1985, 1989). Only if there are very stable and obvious differences in the measured values can such biases be neglected (e.g. in the results of Jordaens *et al*., 2002). We therefore sought qualitative traits (e.g. the basic patterns of plicae in the penis) that are constant even in geographically separated populations.

### Shell morphology

Seven parameters of shell morphology described by Duda *et al.* (2011) were recorded (four qualitative and three quantitative traits). The four quantitative shell traits were measured in intact adult specimens with a graduated eyepiece under a stereomicroscope: shell diameter, umbilicus diameter, shell height and height of last whorl. These values were log_10_ transformed for subsequent analyses. Furthermore, three qualitative aperture traits were recorded: basal tooth (similar to the one of *Petasina unidentata*, see also Duda *et al*., 2011), internal rib and paler area around the aperture. The quantitative measurements were subjected to a discriminant analysis (DA). In the next step, quantitative measurements and qualitative data were merged in a combined DA. For this, the qualitative data were subjected to a correspondence analysis and the first three dimensions of this analysis were added to the matrix (containing the log-transformed measurement values) of the quantitative data (Tabachnik & Fidell, 1996). This combination should separate different groups better and was performed as an operative tool of descriptive statistics. The analyses included (1) individuals of the *T. hispidus/sericeus* complex only and (2) the complete dataset, including individuals of other taxa as well. The software R (R Development Core Team, 2012) was used for all calculations.

In the *T. hispidus* complex the ratios ‘shell width/umbilicus width’, ‘shell width/shell height’ and ‘shell height/height of last whorl’ were also calculated (see Supplementary Material, Table S1). Both ratios and measurements here set in relation to geographic information (elevation and longitude) to test whether they were correlated with those parameters. Therefore, the coefficient of determination was calculated by MS Excel. The ratio ‘umbilicus width/shell width’, as used by Proćków, Mackiewicz & Pieńkowska (2013), was also calculated and compared with our results. Those authors defined values of this ratio of 0.18–0.16 as the overlapping area between *T. hispidus* and *T. sericeus*, and values below 0.16 as exclusively typical for *T. sericeus*. Therefore, we searched for individuals with a relative umbilicus diameter below 0.18 and compared our results with the suggestions of Proćków *et al*. (2013) with regard to clades as well as populations.

### Genital anatomical traits

We followed the approach already used by other authors for *Trochulus* species (Schileyko, 1978, 2006; De Winter, 1990) and produced internal sections of the genital tract, i.e. cross sections of the penis, to record the basic patterns of plicae. Our aim was to compare the results with those from previous studies. Ten individuals of each mt clade were analysed. If fewer individuals were available from a particular clade, all specimens were analysed. Specimens were selected to represent differing regions as much as possible. A total of 108 individuals were dissected. In addition to individuals of the processed species (68 *T. hispidus*, 21 *T. oreinos* subspp. and 10 *T. striolatus* subspp.), single representatives of related taxa (respectively one individual of *T. villosus*, *T. clandestinus* and two individuals of *T. villosulus*, *T. coelomphala* and *Plicuteria lubomirskii*) were also dissected. In the *T. hispidus* complex, 69 adult individuals were included in the anatomical investigation representing the following clades: clade1: 9, clade 2: 20 (2a: 10, 2b: 10), clade 3: 10, clade 5: 1, clade 6: 10, clade 8: 9, clade 9: 10. All specimens were photographed before sectioning.

### Habitat analyses

At the species level, a correspondence analysis (using R software) was performed to evaluate whether habitat parameters such as vegetation type and landscape structure (defined in Tables 3 and 4) revealed different habitat requirements. Only ecological data evaluated by the present authors were used in the analysis. The values of the first two dimensions were visualized in a scatterplot, where factors with the highest impact on these dimensions were highlighted. Raw data are provided in the Supplementary Material (Table S2).

## RESULTS

### Shell morphology

**Figure 3.**
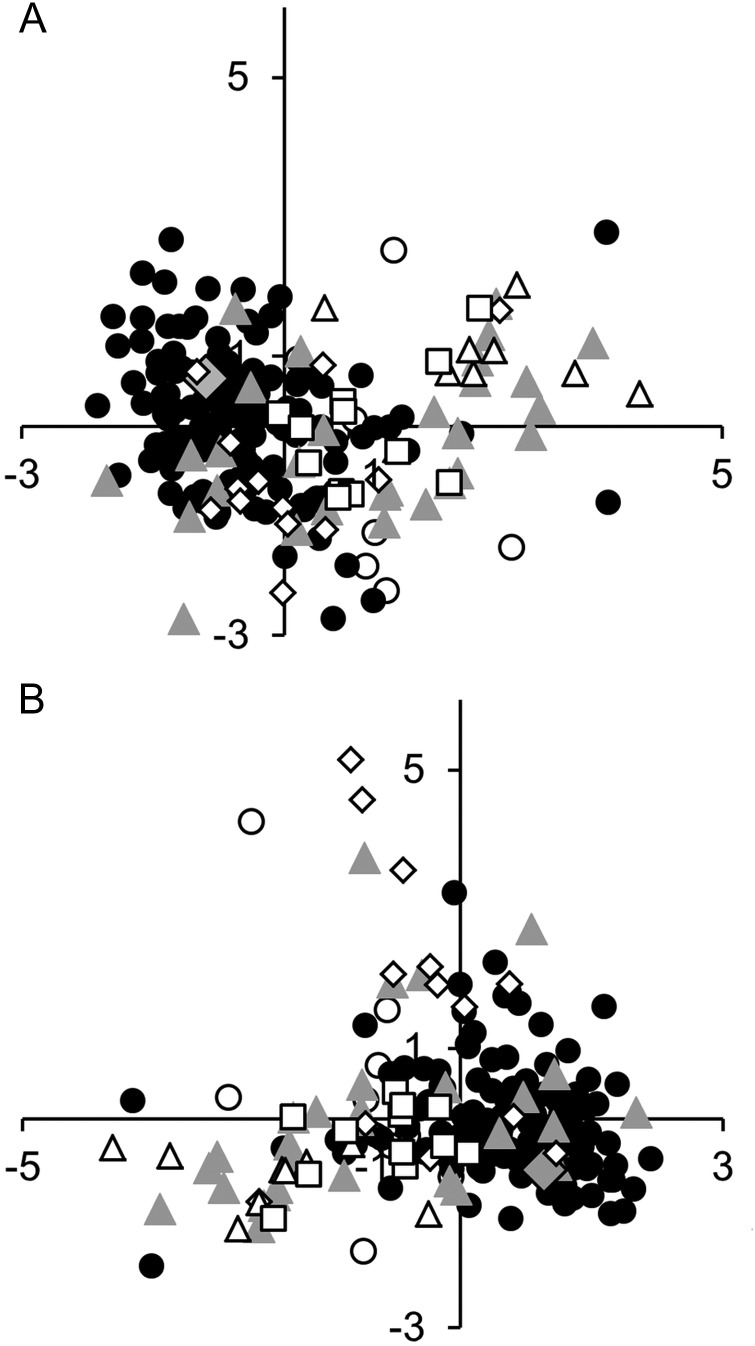
**A.** First two axes of a discriminant analysis of seven clades within the *Trochulus hispidus* complex based on measurements. Symbols: white circles, clade 1; black circles, clade 2; grey triangles, clade 3; grey rhombs, clade 5; white rhombs, clade 6; white triangles, clade 8; white squares, clade 9. LD1 on horizontal axis, LD2 on vertical axis. Coefficients of linear discriminants (LD1, LD2): shell width: −4.18, 41.54; width of umbilicus: −7.48, −10.22; shell height: 14.85, −23.39; height of last whorl: −2.06, −17.71. **B.** First two axes of a combined discriminant analysis of seven clades within the *T. hispidus* complex based on shell measurements and the first three dimensions of a correspondence analysis of qualitative shell traits. Symbols and axes as in **A**. Coefficients of linear discriminants (LD1, LD2): dimension 1: 0.19, −0.65; dimension 2: 0.06, 0.13; dimension 3: 0.27, −0.79; shell width: 5.74, −20.69; width of umbilicus: 7.03, 5.84; shell height: −16.06, 13.78; height of last whorl: 1.65, 6.11.

To evaluate potential differences among mt clades (detected by Kruckenhauser *et al*., 2014) not apparent by visual inspection individuals representing the *Trochulus hispidus* complex were subjected to a morphometric analysis of shell characters. Individuals, raw data and the corresponding clades are listed in Supplementary Material, Table S1. Subsequently, the complete dataset was analysed, including individuals of other taxa as well. Individuals of the *T. hispidus* complex (specifically clades 2, 3, 6 and 9) showed very variable shell measurements largely overlapping between clades (Supplementary Material, Tables S1 and S5). In particular, umbilicus width ranged broadly from 0.4 to 2.5 mm (standard deviation, SD = 0.41). To test statistically this observed lack of differentiation of clades (Table 5) a DA was performed with the individuals of the *T. hispidus* complex; no differentiation was found, either in the DA based on measurement values only (Fig. 3A) or in the combined DA (measurements plus qualitative traits, Fig. 3B). Representatives of all clades form mostly overlapping clouds in the biplot of the first two axes (Table 6).

**Table 5.** Summary of shell measurements (mm) of different *Trochulus* taxa and mt clades.

	SW	WU	SH	HW	SW	WU	SH	HW
T/C	*T. hispidus* all clades (*n* = 212)				*T. hispidus* clade 1 (*n* = 9)			
Range	5.2–9.3	0.4–2.5	2.7–5.5	1.6–3.8	6–7.8	0.7–1.4	3.2–5.0	2.5–3.8
Mean	7.13	1.43	3.92	2.91	6.96	1.12	4.16	3.13
SD	0.88	0.41	0.50	0.34	0.74	0.21	0.60	0.42
SE	0.06	0.03	0.03	0.02	0.25	0.07	0.20	0.14
T/C	*T. hispidus* clade 2 (*n* = 139)				*T. hispidus* clade 3 (*n* = 29)			
Range	5.2–9.1	0.4–2.3	2.7–4.9	1.6–3.8	5.3–9.3	0.5–2.5	3.1–5.0	2.3–3.6
Mean	7.23	1.55	3.85	2.88	6.84	1.22	4.01	2.97
SD	0.76	0.30	0.44	0.34	1.21	0.69	0.57	0.35
SE	0.06	0.03	0.04	0.03	0.26	0.13	0.13	0.08
T/C	*T. hispidus* clade 6 (*n* = 13)				*T. hispidus* clade 8 (*n* = 9)			
Range	5.7–9	0.6–2	3.5–4.7	2.3–3.6	5.3–8.1	0.6–1.1	3.3–5.5	2.6–3.2
Mean	7.60	1.60	4.29	3.04	6.46	0.77	4.01	2.80
SD	1.01	0.40	0.52	0.36	0.91	0.17	0.68	0.17
SE	0.28	0.11	0.14	0.10	0.30	0.06	0.23	0.06
T/C	*T. hispidus clade* 9 (*n* = 12)				*T. oreinos*, both subspp. (*n* = 42)			
Range	5.7–7.9	0.7–1.3	3.2–4.7	2.5–3.3	5.9–7.5	0.9–1.5	2.9–4.1	1.5–2.8
Mean	6.79	1.11	4.00	2.93	6.53	1.20	3.42	2.37
SD	0.59	0.17	0.41	0.20	0.43	0.14	0.32	0.23
SE	0.17	0.05	0.12	0.06	0.06	0.02	0.05	0.04
T/C	*T. o. oreinos* (*n* = 21)				*T. o. scheerpeltzi* (*n* = 21)			
Range	5.9–7.3	0.9–1.4	2.9–4.1	1.5–2.8	5.9–7.5	0.9–1.5	2.9–4.0	2.0–2.7
Mean	6.53	1.23	3.40	2.38	6.52	1.17	3.45	2.36
SD	0.44	0.13	0.36	0.28	0.41	0.15	0.28	0.18
SE	0.10	0.03	0.08	0.06	0.09	0.03	0.06	0.03
T/C	*T. striolatus*, three subspp. (*n* = 26)				*T. s. striolatus* (*n* = 11)			
Range	9.0–13.5	1.3–2.4	4.7–8.4	3.5–5.5	9.0–13.5	1.4–2.4	4.8–8.4	3.5–5.5
Mean	10.71	1.75	6.13	4.45	11.01	1.96	6.37	4.54
SD	1.20	0.36	0.85	0.51	1.54	0.42	1.11	0.63
SE	0.23	0.07	0.17	0.10	0.46	0.13	0.33	0.13
T/C	*T. s. danubialis* (*n* = 7)				*T. s. juvavensis* (*n* = 8)			
Range	9.7–12	1.4–1.9	5.6–6.8	4.3–5.0	9.2–11.3	1.4–2.1	4.7–6.3	3.5–4.8
Mean	11.01	1.71	6.29	4.56	10.04	1.69	5.68	4.24
SD	0.74	0.22	0.41	0.27	2.83	2.83	2.83	2.83
SE	0.28	0.08	0.15	0.10	0.22	0.11	0.18	0.16

Measurement values for all clades (also for those with sample sizes <10) are given to show the whole spectrum of variation (except for clades 4 and 7 of which no adult specimens were available and clade 5 where just one specimen was available). Abbreviations: T/C, taxon/clade; SD, standard deviation; SE, standard error of mean; SW, shell width; WU, umbilicus width; SH, shell height; HW, height of last whorl.

**Table 6.** All sample sites containing Trochulus specimens with a relative umbilicus diameter (umbilicus width/shell width) <1.8.

spID	inID	Alt	C	SW/WU	WU/SW	spID	inID	Alt	C	SW/WU	WU/SW
168	1296	470	2A	4.12	0.243	*455*	*4293*	*110*	*1*	*6.25*	*0.160*
168	1295	470	2A	4.19	0.239	***455***	***4294***	***110***	***1***	***8.33***	***0.120***
*168*	*1294*	*470*	*2A*	*6.00*	*0.167*	***541***	***6250***	***1755***	***8A***	***7.00***	***0.143***
204	1460	1620	2A	5.31	0.188	548	6407	820	2A	5.00	0.200
***204***	***1481***	***1620***	***2A***	***6.64***	***0.151***	548	6235	820	9	5.15	0.194
***204***	***1482***	***1620***	***2A***	***7.00***	***0.143***	548	6237	820	9	5.42	0.185
***215***	***1803***	***1090***	***3A***	***8.83***	***0.113***	***548***	***6405***	***820***	***9***	***6.58***	***0.152***
***215***	***1804***	***1090***	***3A***	***9.17***	***0.109***	***548***	***6236***	***820***	***2A***	***6.64***	***0.151***
***215***	***1802***	***1090***	***2A***	***13.50***	***0.074***	***548***	***6404***	***820***	***2A***	***6.90***	***0.145***
*217*	*1475*	*1048*	*3A*	*5.91*	*0.169*	***548***	***6406***	***820***	***9***	***7.00***	***0.143***
*217*	*1476*	*1048*	*3A*	*6.00*	*0.167*	***548***	***6408***	***820***	***9***	***7.60***	***0.132***
***217***	***1813***	***1048***	***3A***	***6.80***	***0.147***	***548***	***6409***	***820***	***2A***	***7.89***	***0.127***
***217***	***1474***	***1048***	***3A***	***6.82***	***0.147***	***548***	***726***	***820***	***2A***	***8.71***	***0.115***
***217***	***1812***	***1048***	***3A***	***12.00***	***0.083***	*549*	*6413*	*599*	*6A*	*6.20*	*0.161*
231	1836	165	2A	5.50	0.182	***549***	***6411***	***599***	***3A***	***6.55***	***0.153***
*231*	*1834*	*165*	*2A*	*6.17*	*0.162*	***549***	***6410***	***599***	***3A***	***7.00***	***0.143***
***248***	***2079***	***1802***	***8B***	***9.00***	***0.111***	***549***	***6234***	***599***	***6A***	***9.50***	***0.105***
***248***	***2080***	***1802***	***8B***	***10.83***	***0.092***	***549***	***6412***	***599***	***9***	***9.57***	***0.104***
***407***	***4155***	***781***	***3A***	***8.83***	***0.113***	***549***	***6233***	***599***	***3A***	***10.17***	***0.098***
***407***	***4156***	***781***	***3A***	***9.50***	***0.105***	550	6230	600	9	5.18	0.193
***407***	***4157***	***781***	***3A***	***10.00***	***0.100***	550	6416	600	9	5.38	0.186
***412***	***4167***	***671***	***3A***	***8.14***	***0.123***	550	6229	600	9	5.55	0.180
***412***	***4166***	***671***	***3A***	***8.29***	***0.121***	*550*	*6417*	*600*	*9*	*5.62*	*0.178*
***412***	***4165***	***671***	***3A***	***9.17***	***0.109***	*550*	*6415*	*600*	*9*	*6.00*	*0.167*
418	4176	−23	1	5.55	0.180	***550***	***6414***	***600***	***3A***	***6.30***	***0.159***
418	4175	−23	1	5.67	*0.176*	***550***	***6231***	***600***	***9***	***6.40***	***0.156***
***418***	***4177***	−***23***	***1***	***8.57***	***0.117***	***555***	***6248***	***105***	***8A***	***7.75***	***0.129***
*446*	*4264*	*1148*	*2A*	*5.70*	*0.175*	***555***	***6249***	***105***	***8A***	***8.13***	***0.123***
*446*	*4263*	*1148*	*2A*	*5.77*	*0.173*	***556***	***6238***	***100***	***8A***	***8.57***	***0.117***
***446***	***4265***	***1148***	***2A***	***7.13***	***0.140***	***556***	***6240***	***100***	***8A***	***10.17***	***0.098***
451	4285	85	1	5.45	0.183	***561***	***6246***	***2195***	***8A***	***6.63***	***0.151***
*451*	*4286*	*85*	*1*	*6.08*	*0.164*	***561***	***6245***	***2195***	***8A***	***9.50***	***0.105***

Normal text indicates umbilicus diameter >1.8; italic font indicates umbilicus diameter <1.8 to >1.6; bold italic font indicates umbilicus diameter <1.6, according to the results of Proćków, Mackiewicz & Pieńkowska (2013).

It was clearly not possible to distinguish the mt *T. hispidus* clades detected by Kruckenhauser *et al.* (2014) or the problematic taxon *T. sericeus* in the DAs, either based on measurements only or by a combination of measurements and the first three dimensions of a correspondence analysis. The ‘predict’ function of the program R (R Development Core Team, 2012) based on a linear model object, in which we tried to predict the clade affiliation of specimens, also led to a high number (about 40%) of misidentifications in both analyses (measurements alone as well as measurements combined with qualitative traits) in clades 1, 3, 5, 6, 8 and 9. Some clades were even not recognized in the ‘predict’ function using both datasets (measurements and qualitative characters), namely clades 1, 5, 6 and 9. The high recognition number of clade 2 (about 90%) reflects the disproportionally high number of individuals within this clade compared with the other clades. To override this bias, we used trained models with a reduced dataset (R Development Core Team, 2012); however, this attempt also failed to clearly separate the clades. To illustrate the enormous morphological variation within and among clades of the *T. hispidus* complex, photographs of representative shells are compiled in the Supplementary Material together with representatives of *T. striolatus* and *T. oreinos* subspp. (Supplementary Material, Figs S3 and S4).

Representatives of clade 1 (northern Europe), clade 8 (Baden-Württemberg in Germany, Switzerland) and clade 9 (Tirol in Austria) had a narrower umbilicus, while those from other clades showed a broad variability (Table 5 and Supplementary Material, Table S1). All individuals in clades 1 and 8 and 50% of individuals in clade 9 had a shell width/umbilicus width ratio higher than 5.7. Ratios of globularity did not yield clear results, as the clades are spread over the whole range of values. Over the whole sample, there is a moderate correlation of shell measurements and ratios with longitude: Shell width (*R*^2^ = 0.2197) and umbilicus width (*R*^2^ = 0.4243) tend to be smaller towards the west, while the ratio shell width/umbilicus width increases towards the west (*R*^2^ = 0.3151) (Supplementary Material, Table S1). The *R*^2^ values for the height of the last whorl (0.0126) and the ratio shell height/height of last whorl (0.0021), both tending to be bigger in the east, were negligible. Concerning a correlation of shell measurements and sea level, all *R*^2^ correlation coefficients were very low (<0.2) and there was a broad distribution of values. Most values of *R*^2^ were negligible (shell height: *R*^2^ = 0.0156; height of last whorl: *R*^2^ = 0.0154; shell width/shell height: *R*^2^ = 0.022; shell height/height of last whorl: *R*^2^ = 0.0005). The ‘highest’ *R*^2^ were found for the width of umbilicus and the ratio shell width/width of umbilicus, becoming smaller with increasing sea level (*R*^2^ = 0.0824 and *R*^2^ = 0.0626, respectively) and the shell width becoming larger at lower elevations (*R*^2^ = 0.0579). This is a (of course weakly) supported hint that the narrowness of the umbilicus is somehow associated with higher elevations. It has to be mentioned that both factors are interconnected concerning our sample sites, i.e. sample sites in the west are in most cases located at higher elevations than those in the east. This phenomenon is observed within clades 2, 3 and 6. An exception can be seen in clade 8: here four individuals with a very narrow umbilicus are also found at low altitudes in the sample sites 555 and 556. However, it has to be emphasized that these are single individuals and the sample size is small.

**Figure 4.**
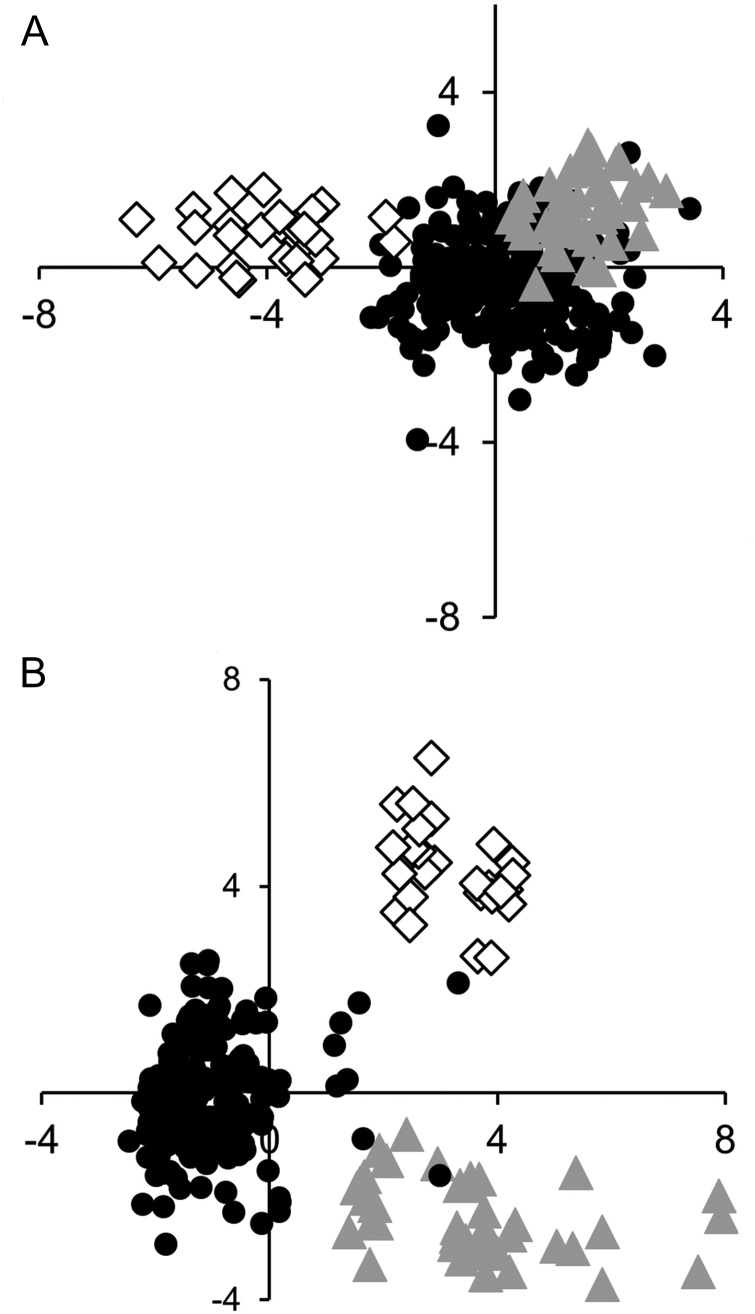
**A.** First two axes of a discriminant analysis of three *Trochulus* species based on measurements. Symbols: black circles, *T. hispidus* complex; white rhombs, *T. striolatus* subspp.; grey triangles, *T. oreinos* subspp. LD1 on horizontal axis, LD2 on vertical axis. Coefficients of linear discriminants (LD1, LD2): shell width −10.66, 52.96; width of umbilicus: 2.43, −12.12; shell height: −0.69, −11.39; height of last whorl: −13.33, −26.36. **B.** First two axes of a combined discriminant analysis of the three species based on measurements and the first three dimensions of a correspondence analysis of qualitative shell traits. Symbols and axes as in **A**. Coefficients of linear discriminants (LD1, LD2): dimension 1: −1.84, 0.61; dimension 2: 0.57, 0.29; dimension 3: −0.85, −0.26; shell width: 18.93, 11.88; width of umbilicus: −4.85, −0.59; shell height: −4.75, 2.33; height of last whorl: −5.08, 9.64.

The morphometric analysis including related taxa (*T. striolatus* subspp., *T. oreinos* subspp.) revealed *T. striolatus* and *T. oreinos* subspp. as partly separated in the analysis based just on measurements (Fig. 4A), as the clouds of especially the *T. hispidus* complex and *T. oreinos* overlapped. This led to a misidentification of 10% (28/280) of the investigated specimens in the ‘predict’ function of R (8 *T. hispidus* identified as *T. oreinos*, 18 *T. oreinos* as *T. hispidus* and 2 *T. striolatus* as *T. hispidus*).

The combined DA of measurements and the first three dimensions of qualitative characters led to a better separation. Here the ‘predict’ function showed clear separation of the three groups. There was only one outlier of the *T. hispidus* complex that was predicted to be a member of *T. oreinos* in the analysis based on measurements (see also Fig. 4B).

**Figure 5.**
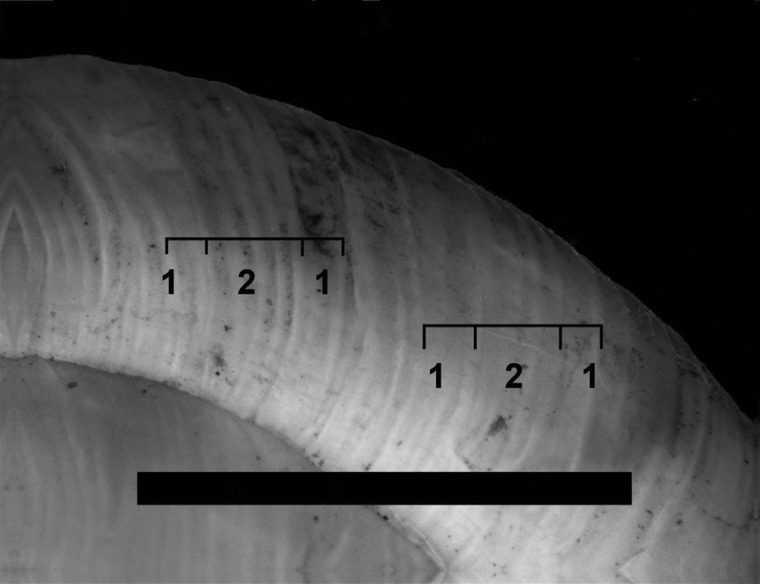
Characteristic riffle structures on the periostracum of *Trochulus striolatus* subspp., illustrated by an individual of the nominate subspecies (individual no. 4043, sample site no. 416); coarse ribs (1; spacing about 0.5 mm) are followed by narrow ones (2; spacing smaller than 0.25 mm). Scale bar = 5 mm.

In *T. striolatus*, the occurrence of ‘double riffles’ and fields of coarse ribs (spacing about 0.5 mm) followed by smooth ones (spacing smaller than 0.25 mm) appeared to be a discriminating trait separating it from the *T. hispidus* complex (Fig. 5). Within *T. striolatus* there were only subtle shell morphological differences between the nominate form and the subspecies *T. s. danubialis* on one hand and the subspecies *T. s. juvavensis* on the other. The latter appeared to be smaller (Table 5). Small sample size, however, precludes conclusive statements.

### Anatomical analyses

**Figure 6.**
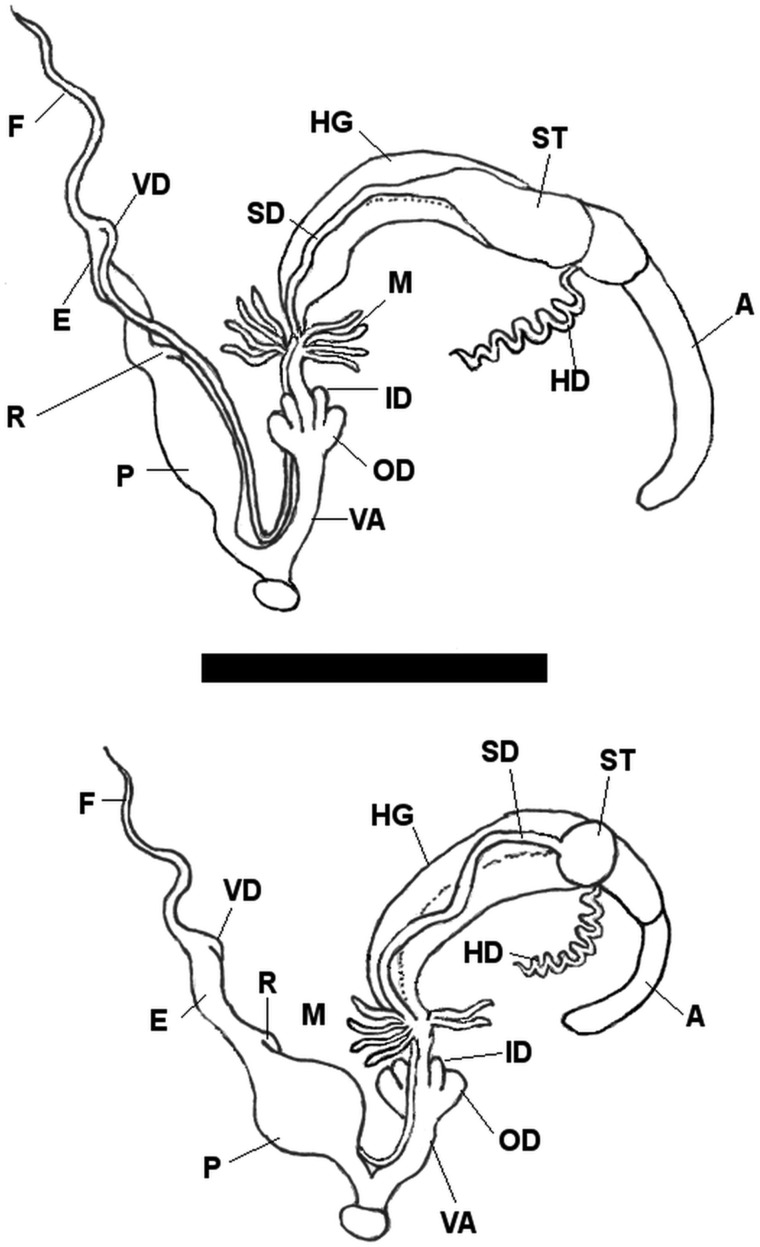
Two variants of *Trochulus hispidus* genitalia. The upper one shows a fusiform penis, elongate spermatheca and four pairs of mucous glands, the lower one a bulbous penis, round spermatheca and three pairs of mucous glands. Abbreviations: A, albumen gland; E, epiphallus; F, flagellum; HD, hermaphroditic duct; HG, hermaphroditic gland; ID, inner dart sacs; M, mucous glands; OD, outer dart sacs; P, penis; R, retractor muscle; SD, spermathecal duct; ST, spermatheca; VA, vagina; VD, vas deferens. Scale bar = 5 mm.

In the next step, representatives of different clades and described taxa were investigated with respect to differences in genital anatomy. Among representatives of clades of the *T. hispidus* complex, no constant differences were found in the shape of the bursa copulatrix, penis form or flagellum length; all these traits showed high variability (two pronounced variations are shown in Fig. 6). In particular, individuals with a relatively narrower umbilicus are not conspicuous in their genital anatomy.

**Figure 7.**
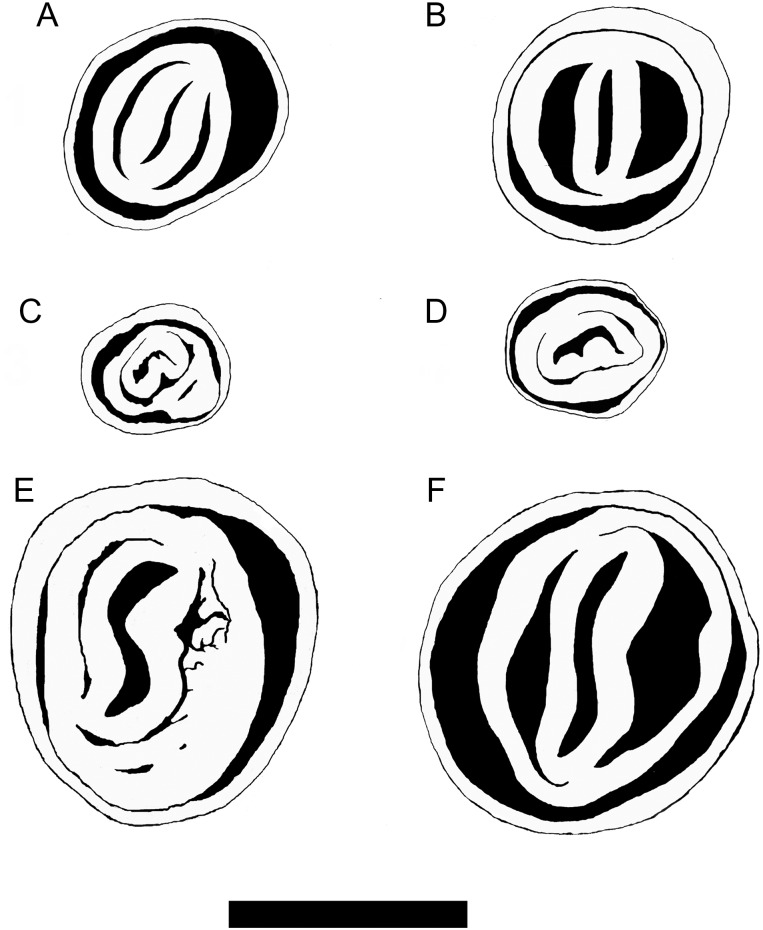
Ground patterns of penis cross section in the *Trochulus hispidus* complex, *T. striolatus* subspp. and *T. oreinos* subspp. **A**. *T. hispidus* complex with small folds. **B**. *T. hispidus* complex with broad folds. **C**. *T. oreinos* with additional fold and bulge (only found in *T. o. oreinos*). **D**. *T. oreinos* with no additional fold (only found in *T. o. scheerpeltzi*). **E**. *T. striolatus* with folds with protuberances (mainly found in *T. s. striolatus*). **F**. *T. striolatus* with smooth folds (found in *T. s. danubialis* and *T. s. juvavensis*)*.* Scale bar = 1 mm.

Moreover, the consistently spherical (i.e. as long as broad) spermatheca—described as a typical trait of *T. sericeus* in Great Britain and mainland France by Anderson (2005)—could not be verified in our material. The presence of three instead of four pairs of mucous glands (Fig. 6), which was reported to be a discriminating trait for the poorly described and disputed taxon *T. suberectus*, occurred just occasionally in clades 2 (subclade 2b; 1 out of 10), 8 (3 of 9) and 9 (1 of 10). The pattern of folds in the cross section of the penis showed no variation in the *T. hispidus* complex (Fig. 7), whereas the diameter varied somewhat.

In contrast, the related species can be distinguished by specific differences in their genital anatomy, i.e. in the penis structure observed in cross section. In *T. oreinos* the penis has a single intrapapillar cavity interrupted at one side (Fig. 7). One constant difference was detected between the two *T. oreinos* subspecies: *T. o. oreinos* has a bulge attached to the penial fold, which occasionally has an additional small fold, whereas *T. o. scheerpeltzi* lacks this trait (Fig. 7C, D). *Trochulus striolatus* could be distinguished from *T. hispidus* in some cases by a penis with additional folds or modified folds with protuberances (Fig. 7E, F). Nevertheless, in all seven specimens of *T. striolatus*, representing the subspecies *danubialis* and *juvavensis*, the arrangement of the penial folds was the same as in *T. hispidus*. Thus, this structure seems to be very variable in *T. striolatus*.

**Figure 8.**
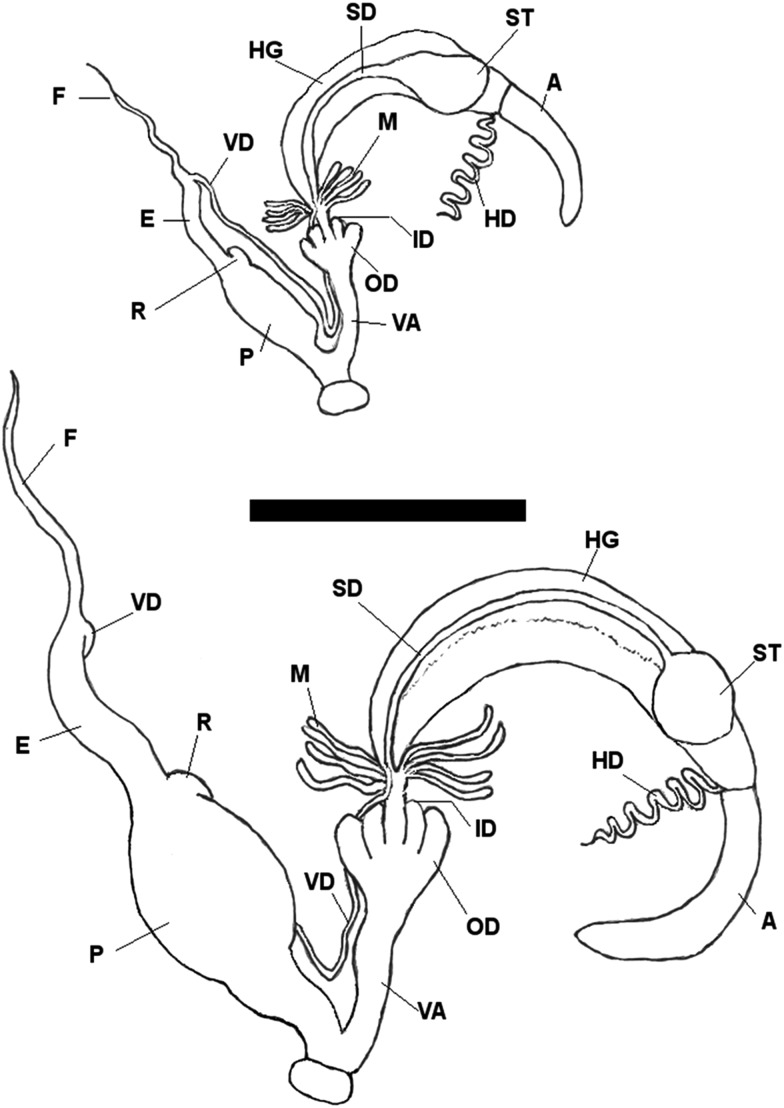
Genital duct of *Trochulus oreinos* (top) and *T. striolatus* (bottom). Abbreviations: A, albumen gland; E, epiphallus; F, flagellum; HD, hermaphroditic duct; HG, hermaphroditic gland; ID, inner dart sacs; M, mucous glands; OD, outer dart sacs; P, penis; R, retractor muscle; SD, spermathecal duct; ST, spermatheca; VA, vagina; VD, vas deferens.

Besides these specific traits, the general genital anatomy of *T. oreinos*, *T. striolatus* and *T. hispidus* showed no constant differences. Examples of the genital duct and cross sections of the penis of the various taxa are shown in Figures 7, 8 and in the Supplementary Material (Figs S5–S8).

### Identification of other species

The identifications of *T. villosus*, *T. villosulus*, *T. clandestinus*, *T. biconicus* and *Plicuteria lubomirskii* were straightforward based on the shell morphological and anatomical traits described by Ložek (1956), Kerney *et al.* (1983) and Proćków (2009). *Trochulus coelomphala* proved to be problematic because two representatives of its clade resembled the *T. hispidus* morphotype, while the other three specimens from Günzburg showed the expected *T. coelomphala* morphotype, i.e. a broad umbilicus (umbilicus width about a quarter of total shell width) and a slender upper vagina (details shown in Supplementary Material, Figs S9 and S10).

### Habitat analyses

**Figure 9.**
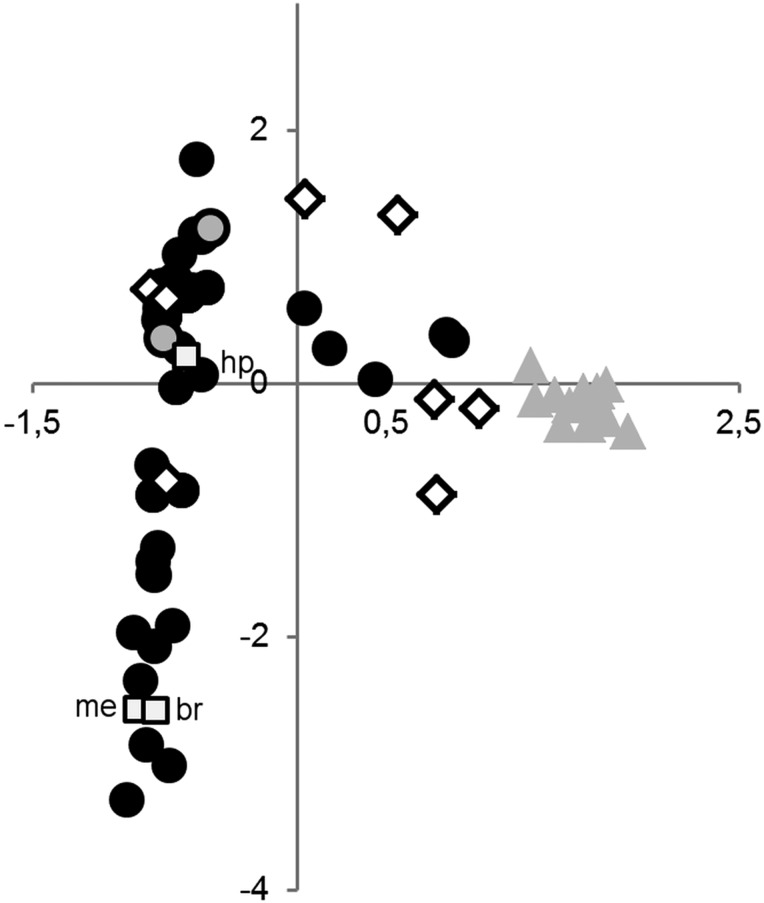
Correspondence analysis based on habitat types and landscape structures of 86 sample sites: biplot of the first two dimensions (horizontal axis is dimension 1, vertical axis is dimension 2). Symbols: black circles, sample sites of *T. hispidus* complex (*n* = 57); grey circles, sample sites with co-occurrence of *T. hispidus* complex and *T. striolatus* subspp. (*n* = 2); white rhombs, sample sites of *T. striolatus* subspp. (*n* = 8); grey triangles, sample sites of *T. oreinos* subspp. (*n* = 19); grey squares, habitat types and landscape structures with highest impact on first two dimensions. Abbreviations: hp, high perennial herbs; br, boundary ridge; me, meadow.

In a correspondence analysis, we tested which taxa were separated according to their ecological preferences (for habitat and landscape structures see Tables 3 and 4). This analysis showed a clear separation of *T. oreinos* from *T. hispidus* and *T. striolatus* (Fig. 9)*.* The localities of the latter two species occupied a large space in the plot, with widely overlapping clouds and only a few sample sites lying close to the cloud representing localities of *T. oreinos*. This configuration reflects the broad ecological niche of *T. hispidus* and *T. striolatus*, which inhabit a wide variety of habitats, whereas *T. oreinos* is an inhabitant of rocky alpine sites. The values responsible for separating *T. oreinos* from the two other taxa are ‘rocks’, ‘boulders’, ‘free of vegetation’, ‘*Pinus mugo* shrubbery’ and ‘(sub)alpine meadows’. The space occupied by *T. hispidus* and *T. striolatus* is vaguely differentiated, but still widely overlapping. The cloud on the positive side of the first dimension represents mainly alpine or rocky habitat (dominant factors: rocks, boulders and alpine grassland), the other one located on the negative side represents the remaining habitats (dominant factors: high perennial herbs, meadow and boundary ridge). Additionally, the *T. hispidus* complex and *T. striolatus* subspp. tend to occur preferentially near to water bodies; this is the case at 44 of the 60 sample sites with individuals of the *T. hispidus* complex and six of 10 sites with records of *T. striolatus*, but only at one of 19 sites with records of *T. oreinos* subspp. Among the clades of the *T. hispidus* complex, no differences were detected with regard to ecological preferences.

## DISCUSSION

### Variation within the *Trochulus hispidus* complex

The clades of the *T. hispidus* complex were separated from each other by unexpectedly high genetic distances ranging up to 18.9% (p distances of COI sequences; Kruckenhauser *et al*., 2014). Nevertheless, they could not be differentiated based on the morphological and anatomical characters investigated. The highly variable shell morphology—even within populations—supports the results of Proćków (2009). In view of this, and with no information about gene flow, the taxonomic status of the clades of the *T. hispidus* complex remains debatable and some of these clades might represent cryptic species. Yet, as long as no unequivocal evidence for the species status of these clades exists, they should be considered as members of a single species. This approach has been used by Pinceel *et al.* (2004), who found highly divergent mt clades within the slug *Arion subfuscus* but treated them as one species because there were no morphological traits to separate them. Concerning the definition of *T. sericeus* by the relative width of umbilicus according to Proćków *et al*. (2013), all clades (except clade 8) in our study that included the *T. sericeus* morphotype (relative umbilicus width <1.6) also included specimens with intermediate (1.6–1.8) or broad umbilicus assigned to *T. hispidus* (>1.8). Considering populations, a similar picture is observed. Clade 8 is the only one in which relative umbilicus width and genetic affiliation are consistent. Our results are mostly in accordance with those of Naggs (1985) and Proćków (2009), who were not able to delimit this taxon. On the other hand, preliminary results from the Czech Republic indicate a separation of *T. sericeus* from two clades of *T. hispidus* in Bohemia and Moravia (Hrabáková*,* Juřičková & Petrusek, 2006; *T. sericeus* assigned as *T. plebeius* by these authors). Moreover, Juřičková & Ložek (2008) reported both species to be parapatric in the Czech Krkonoše mountains and, according to M. Horsák and L. Jurickova (personal communication), Czech populations of *T. hispidus* and *T. sericeus* can be separated straightforwardly. Ložek (1963) also enumerated some descriptive traits, including an elliptic peristome and a tendency for longer hair (average length 0.5 mm). Perhaps a more detailed study on extensive Czech *Trochulus* material would bring new insights to the *hispidus/sericeus* problem. As long as we do not have a comprehensive tree of mtDNA including presumed *T. sericeus* from the Czech Republic and tentatively determined *T. sericeus* specimens (investigated by Proćków *et al*., 2013), it remains open if clade 8 represents the ‘real’ *T. sericeus* or not. Moreover, the small number of our sample (nine individuals) has to be considered.

*Trochulus suberectus*, another poorly described taxon, could not be confirmed by our results. As mentioned in the anatomical analysis, the occurrence of three instead of four pairs of mucous glands, which is the discriminating trait for this dubious species (Proćków, 2009), occurred occasionally in several clades. This observations support Turner *et al.* (1998), who placed *T. suberectus* in the synonymy of *T. sericeus*.

Concerning *T. coelomphala*, the present data are insufficient to decide whether it is an independent species or a subspecies of *T. hispidus.* Kruckenhauser *et al.* (2014) tentatively assigned five individuals forming a separate clade to this taxon based on their geographic origin. In the present study they were not tested as a separate group due to the small sample size of five individuals from two localities. Three of them correspond to the ‘classical’ morphotype of *T. coelomphala*, because they resemble the comparably large (shell width >8 mm), flat *Trochulus* morph with a very broad umbilicus. Moreover, they were collected near Günzburg, a locality well known for this form (Falkner, 1973). However, two specimens originating from Regensburg in northern Bavaria resembled a typical *T. hispidus* morphotype (see also photographs in Supplementary Material, Fig. S9)*.* There are three possible explanations for these results (which remain preliminary due to the small sample size): (1) *T. coelomphala* displays a high phenotypic variation similar to that observed in *T. hispidus*. (2) The two specimens are the result of hybridization or introgression. (3) *Trochulus coelomphala* is not a separate taxon, but merely represents another lineage of the highly variable *T. hispidus* complex. Additionally, there is some confusion concerning the French populations comprising very flat *Trochulus* sp. with broad umbilicus from the Rhone valley. This form has sometimes been assigned to *T. coelomphala* (e.g. by Falkner, 1989). In any case, further investigations of *T. coelomphala* are urgently required.

### Differentiation of *T. striolatus* and *T. oreinos*

The differentiation of *T. striolatus, T. oreinos* and the *T. hispidus* complex was straightforward by means of constant diagnostic traits. In addition, some characters such as shell measurements sometimes allowed separation of the species based on trend, although there were overlaps. The status of the Austrian endemic *T. oreinos* as a separate species has already been confirmed by shell morphological, genetic and ecological analyses (Duda *et al*., 2010, 2011; Kruckenhauser *et al*., 2014). The present study found the cross section of the penis to be an additional stable character of *T. oreinos*; its pattern is totally different from that in the *T. hispidus* complex, but quite similar to *T. biconicus* (see also Proćków, 2009). Concerning the two subspecies of *T. oreinos* (*T. o. oreinos* and *scheerpeltzi*), their overlapping shell traits have already been shown in a more extensive dataset (Duda *et al*., 2011). The present study detected a small but constant anatomical difference in the cross section of the penis. These findings are interesting in comparison with the clades of the *T. hispidus* complex; they are genetically divergent to a similar or even higher degree, but could be differentiated neither in conchological characters nor in genital anatomical traits. We assume that the two subspecies of *T. oreinos* evolved independently in isolation over a long period; the genetic data indicate that each underwent bottlenecks (Duda *et al*., 2011; Kruckenhauser *et al*., 2014).

*Trochulus striolatus* is clearly differentiated from the *T. hispidus* complex by its specific riffle pattern on the shell surface and its genetic traits. Other morphological or anatomical traits such as shell measurements, structure of genitalia or of penial plicae separated only some individuals from the *T. hispidus* complex. Moreover, the bulky penis was not a constant trait in *T. striolatus*, as claimed by Schileyko (1978) and Proćków (2009). At least one individual in our material (4011 in Supplementary Material, Fig. S7), which had a fusiform penis, suggests that this trait might be more variable. Similar difficulties in separating *T. striolatus* from the *T. hispidus* complex were pointed out by Naggs (1985) and Turner *et al*. (1998). Comparing our data with those of Pfenninger *et al.* (2005), we conclude that among the *striolatus* lineages reported in that study, only lineage A corresponds to *T. striolatus* as defined in our genetic analysis (Kruckenhauser *et al*., 2014). The *T. striolatus* clade in our tree covered a wide geographic area from southwestern Germany to eastern Austria and contained individuals unambiguously determined as *T. striolatus* according to the description above*.* Concerning infraspecific classification, some authors have suggested that subspecies should not be accepted within *T. striolatus* (Anderson, 2005; Proćków, 2009). For the areas investigated, at least the separation of *T. s. striolatus* from the other two subspecies (*T. s. danubialis* and *T. s. juvavensis*) seems to be supported by a subtle anatomical difference: an additional penial plica (see Supplementary Material, Fig. S8). Furthermore, *T. s. juvavensis*, which is geographically restricted to the Salzkammergut area in the northern calcareous Alps in Austria, was characterized by smaller shell dimensions (see Supplementary Material, Fig. S4 and Table 5). In the genetic analysis it was not clearly differentiated from *T. s. danubialis*, while *T. s. striolatus* appeared in two distinct lineages well separated from the other two subspecies. Nevertheless, for further infraspecific taxonomic considerations the sample size and the density of the geographic sampling clearly have to be increased.

### Problems of morphological determination, character selection and species delimitation

The detection of diagnostic traits is important to distinguish species. Shell measurements can be ambiguous in discriminating land-snail species in general, as they may be affected by environmental conditions such as climate and nutrition (Davies, 2004). Nevertheless, a few species can only be separated based on shell measurements, e.g. *Pupilla pratensis* from *P. muscorum* (Horsák *et al*., 2010). Nonetheless, land pulmonates are sometimes defined by weak discriminators even in field guides (e.g. Kerney *et al*., 1983; Falkner, 1989) with descriptions such as ‘umbilicus a little more narrow than’ or ‘shell more slender than’. While skilled malacologists are able to determine taxa based on trends, such descriptions may confuse less experienced persons and lead to incorrect determinations. Therefore, beyond detecting genetically distinct entities, whether such entities can be correlated with morphologically or anatomically differentiated groups is crucial. A major question for the present study was whether taxa and/or clades can be distinguished by morphometric analyses of such characters. For example, several species could be clearly classified morphologically and they were distinctly differentiated in the genetic tree: *T. biconicus*, *T. clandestinus*, *T. oreinos, T. striolatus, T. villosus*, *T. villosulus* and *Plicuteria lubomirskii*. These species can be unambiguously determined by combining shell morphology and anatomical characters (compare the photos in Supplementary Material, Figs S9–S11 with figures of Kerney *et al*., 1983 and Proćków, 2009). However, *T. sericeus* and *T. coelomphala* and the whole *T. hispidus* complex remained problematic.

Another point we underline here is that investigations (qualitative or quantitative) of animals from only a few localities have very limited taxonomic value. Moreover, the use of measurements alone without discriminating qualitative traits can lead to ambiguous results. For example, Naggs (1985) pointed out the case of a British *Trochulus* population whose shell and genitalia dimensions were intermediate between *T. hispidus* and *T. striolatus*. The first attempts in the direction of diagnostic values in *Trochulus* were made by Schileyko (1978, 2006), but his studies often included only few specimens; intraspecific variation could therefore not be recognized, as recognized by the author himself. Similarly, statements by Klöti-Hauser (1920) that there are major differences in genital measurements between *T. hispidus* and related species must be interpreted with caution, because those data are based only on single or very few sampling sites. The variation in shell dimensions within populations as well as within mt clades of the *T. hispidus* complex is extremely high. This necessitates including individuals from many localities, covering the whole distribution area, to search for stable traits. In this respect, even our comprehensive data are preliminary because they are concentrated on Austria and surrounding regions. Nonetheless, the data available on populations outside Austria (this study as well as those of Pfenninger *et al*., 2005 and Kruckenhauser *et al*., 2014) strongly support that our results are representative for the *T. hispidus* complex in general. Still, a multinational mapping project with intense sampling of *T. hispidus* over the whole distribution area is needed to complement the available data and to assess the status of related problematic taxa (e.g. *T. coelomphala*, *T. plebeius* and *T. sericeus*).

It remains open whether (or which of) the clades of the *T. hispidus* complex represent species or not. The issue of potential cryptic species within the *T. hispidus* complex should be addressed by testing for hybridization barriers and gene flow. This could be accomplished by studying reproduction biology and by breeding experiments, as well as by genetic analyses of nuclear markers. The *T. hispidus* complex exemplifies the problematic practice of DNA barcoding without detailed knowledge of phylogenetic/phylogeographic relationships and species delimitation. Even for a comparably small area like the eastern Alps and adjacent regions, a few COI sequences for defining *T. hispidus* are clearly misleading (see also Kruckenhauser *et al*., 2014).

### Phylogenetic and phylogeographic implications

Besides pointing at possibilities and problems of species delimitations, the grouping in the genetic tree of Kruckenhauser *et al.* (2014) shows a big clade of ‘*Trochulus* s. str.’, which is divided into two geographic subclades (Fig. 1): an eastern subclade comprising clades 1–7 and 9, as well as *T. coelomphala*, *T. villosulus* and *T. striolatus*, and a western one consisting of clade 8 as well as *T. clandestinus* and *T. villosus*. Three taxa apparently belong neither to the eastern nor to the western group of ‘*Trochulus* s. str.’: *Plicuteria lubomirskii* (designated as *T. lubomirskii* by some authors, e.g. Proćków, 2009), *T. biconicus* and *T. oreinos*. This agrees with the views of Schileyko (1978), Falkner (1982) and Turner *et al.* (1998), who considered *P. lubomirskii*, *T. oreinos* and *T. biconicus* to be only distantly related to *Trochulus sensu stricto*. Conspicuously, those taxa show either extremely short hairs <0.1 mm (evident in *P. lubomirskii* and *T. oreinos*, see also Proćków, 2009; Duda *et al*., 2011) or no hairs at all (*T. biconicus*). This lends plausibility to Proćków (2009), who considered short hairs or the general lack of hairs on the periostracum within the tribe Trochulini as a plesiomorphic trait, because all the mentioned taxa branch off from basal nodes in the genetic tree. But these implications are only preliminary because final conclusions or a taxonomical review of European Trochulini require more data on all known taxa including the (sub)genera *Petasina* and *Edentiella*. We can, however, definitively reject a possible sister-group relationship of the *T. hispidus* complex with both *T. oreinos* subspp., an issue left unresolved by Duda *et al.* (2011).

### Ecological differences and distribution

Our results show that the *T. hispidus* complex and *T. striolatus* tolerate a wide range of habitats, some of which even come close to the niche of *T. oreinos.* This, however, is true only if the data are based on a few simple categories. With a more detailed analysis including vegetation associations, it is possible to separate *T. oreinos* unambiguously from the others. This confirms our earlier study (Duda *et al*., 2011) in which *T. o. oreinos* was characterized as an inhabitant of cool dry *Caricetum firmae* meadows and boulders with sparse vegetation. A more detailed analysis including Ellenberg values might show more pronounced differences in the habitat needs of the three taxa by characterizing quantitative biotic and abiotic factors (see also Horsák *et al*., 2007).

For the *T. hispidus* complex in the investigated area, the western populations in mountainous regions inhabit habitats slightly different from the eastern lowland populations. The former are less confined to sites adjacent to water bodies and often found at sites without high perennial herbs, but instead on rocks and in subalpine meadows. This may reflect climatic conditions, as the Atlantic climate in the west is more humid. Populations in the eastern Austrian flatlands are strictly bound to wetlands adjoining water bodies. Čejka, Horsák & Némethová (2008) reported similar results for land snail faunas in the Danubian floodplain forests of Slovakia, showing that *T. hispidus* has a moister and *T. striolatus* a drier optimum. In general, members of the *T. hispidus* complex inhabit a broad range of often dynamic or anthropogenically influenced habitats associated with rivers and wetlands. This promotes dispersal, either actively (along river valleys acting as corridors) or passively (drift by flood or anthropogenic transport). In addition, the broad range of possible habitats and the tolerance of different climatic conditions might explain the high variation in morphological and genetic characters and the extensive range of the *T. hispidus* complex, reaching from the northern parts of the Mediterranean peninsulas to Scandinavia and even extending to the colonization of North America as a neobiont (see, e.g. Hotopp *et al*., 2010). This also implies that populations survived several climatically suboptimal periods in various refugia, followed by expansion during warm interglacial periods during the Pleistocene.

In contrast, *T. oreinos* obviously has an entirely different evolutionary history. According to Duda *et al.* (2010), it is a stenoecious inhabitant of a narrow ecological niche consisting of cool, primarily treeless and slightly azonal habitats such as boulders, rocks and *Caricetum firmae* meadows with patchy structure. Such suitable habitats exist all across the northern calcareous Alps, although only a small, restricted area is populated, probably corresponding to habitats that remained ice-free during the last glaciation (Van Husen, 1997). Thus, *T. oreinos* obviously has very restricted dispersal and colonization abilities. In summary, all these factors led to a comparably low genetic and morphological variation within each *T. oreinos* subspecies, which has been further reduced by bottleneck effects (Duda *et al*., 2011).

Compared with the former two species, *T. striolatus* seems to have an intermediate position: it is variable in habitat choice and morphology, but quite homogeneous in mt variation. This might reflect rapid dispersal from a single refugium (or only a few refugia) over large parts of Europe after the last glaciation. At this point our results should also be compared with the hypothesis of prime species and remnant species proposed by Gittenberger & Kokshoorn (2008). In our case, *T. hispidus* and *T. striolatus* would be classified as two phylogenetically divergent forms (high genetic diversity in *hispidus vs* low one in *striolatus*) of a widespread, euryoecious prime species and *T. oreinos* as a stenoecious, geographically restricted remnant species.

### Applied aspects

Irrespective of taxonomic status and of morphological and genetic variation, however, the geographic distribution of clades and morphotypes is relevant from the conservation perspective. The habitats of some clades within the *T. hispidus* complex and several local populations of *T. striolatus* are under pressure. Two regions impacted by landscape degradation should be pointed out. (1) Wetlands and even the big riverine forests in the northern and very eastern flatlands of Lower Austria were heavily influenced by intensive agriculture, construction activity and hydraulic engineering in the last decades of the 20th century. As these habitats are the only ones in which both the *T. hispidus* clade 6B and *T. striolatus danubialis* occur, both taxa might be affected by such anthropogenic impact. The latter taxon is even classified as ‘critically endangered’ in the Red Data Book of Austria (Reischütz & Reischütz, 2007). (2) The inner-alpine valleys of Tyrol and Salzburg are under heavy pressure from settlement development due to the reduced space on the valley plains. Therefore, suitable habitats such as moist meadows have already become extremely rare. This concerns populations of clades 3A and 9. *Trochulus sericeus* and *T. hispidus* (assigned as separate species by Reischütz & Reischütz, 2007) are classified as of ‘least concern’ in the current Red Data Book of Austria, with slight tendencies of decline. Nevertheless, even if none of the clades represents a cryptic species, the extinction of geographically restricted clades would heavily affect intraspecific diversity. Therefore, new conservation policies are required that also protect phylogenetically diverged clades irrespective of their taxonomic status, such as the concept of evolutionarily significant units (Fraser & Bernatchez, 2001).

The existence of many different mt clades in the *T. hispidus* complex and the lack of diagnostic traits with which to differentiate them reveal general problems and limitations of classical (morphology-based) taxonomy in land snails, especially in so-called ‘critical taxa’. Nevertheless, our morphological analyses, together with habitat data, provide valuable information about the morphological and genetic plasticity of the *T. hispidus* complex*.* Moreover, our analyses have yielded important insights in habitat requirements of the species investigated and revealed several new diagnostic traits for interspecific separation as well as for some subspecies of *T. striolatus* and *T. oreinos*.

## SUPPLEMENTARY MATERIAL

Supplementary material is available at *Journal of Molluscan Studies* online.

## References

[EYU023C1] ANDERSON R. (2005). An annotated list of the non-marine Mollusca of Britain and Ireland. Journal of Conchology.

[EYU023C2] ČEJKA T., HORSÁK M., NÉMETHOVÁ D. (2008). The composition and richness of Danubian floodplain forest land snail faunas in relation to forest type and flood frequency. Journal of Molluscan Studies.

[EYU023C3] CLESSIN S. (1874). Die Gruppe *Fruticicola* Held des Genus *Helix* L. Jahrbücher der Deutschen Malakozoologischen Gesellschaft.

[EYU023C4] DAVIES G.M. (2004). Species check-lists: death or revival of the nouvelle école?. Malacologia.

[EYU023C5] DÉPRAZ A., HAUSSER J., PFENNINGER M. (2009). A species delimitation approach in the *Trochulus sericeus/hispidus* complex reveals two cryptic species within a sharp contact zone. BioMedCentral Evolutionary Biology.

[EYU023C6] DE WINTER A.J. (1990). Little known land snails from the French Alpes (Pulmonata). Basteria.

[EYU023C7] DUDA M., KRUCKENHAUSER L., HARING E., SATTMANN H. (2010). Habitat requirements of the pulmonate land snails *Trochulus oreinos oreinos* and *Cylindrus obtusus* endemic to the Northern Calcareous Alps, Austria. Ecomont.

[EYU023C8] DUDA M., SATTMANN H., HARING E., BARTEL D., WINKLER H., HARL J., KRUCKENHAUSER L. (2011). Genetic differentiation and shell morphology of *Trochulus oreinos* (Wagner, 1915) and *Trochulus hispidus* (Linnaeus, 1758) (Pulmonata: Hygromiidae) in the northeastern Alps. Journal of Molluscan Studies.

[EYU023C9] EMBERTON K.C. (1985). Seasonal changes in the reproductive gross anatomy of the land snail *Triodopsis tridentata tridentata* (Pulmonata: Polygyridae). Malacologia.

[EYU023C10] EMBERTON K.C. (1989). Retraction/extension and measurement error in a land snail: effects on systematic characters. Malacologia.

[EYU023C11] FALKNER G. (1973). Studien über *Trichia* Hartmann, I. *Trichia* (*Trichia*) *graminicola* n. sp. aus Südbaden (Gastropoda: Helicidae). Archiv für Molluskenkunde.

[EYU023C12] FALKNER G. (1982). Zur Problematik der Gattung *Trichia* (Pulmonata, Helicidae) in Mitteleuropa. Mitteilungen der Deutschen Malakologischen Gesellschaft.

[EYU023C13] FALKNER G., Fechtner R., Falkner G. (1989). Binnenmollusken. Weichtiere.

[EYU023C14] FALKNER G. (1991). Vorschlag für eine Neufassung der Roten Listen der in Bayern vorkommenden Mollusken (Weichtiere). Mit einem revidierten systematischen Verzeichnis der in Bayern nachgewiesenen Molluskenarten. Schriftenreihe des Bayerischen Landesamt für Umweltschutz.

[EYU023C15] FALKNER G. (1995). Beiträge zur Nomenklatur europäischer Binnenmollusken, VII. Nomenklaturnotizen zu europäischen Hygromiidae (Gastropoda: Stylommatophora). Heldia. Münchner malakologische Mitteilungen.

[EYU023C16] FALKNER G., BANK R.A., VON PROSCHWITZ T. (2000). Check-list of the non-marine molluscan speciesgroup taxa of the states of Northern, Atlantic and Central Europe (CLECOM I). Heldia. Münchner malakologische Mitteilungen.

[EYU023C17] FALKNER G., RIPKEN T.H.E., FALKNER M. (2002). Mollusques continentaux de France. Liste de référence annotée et bibliographie. Collection Patrimoines Naturels.

[EYU023C18] FOCART L. (1965). New researches on *Trichia hispida* (Linnaeus) and related forms. Proceedings of the First Malacological Congress.

[EYU023C19] FRASER D.J., BERNATCHEZ L. (2001). Adaptive evolutionary conservation: towards a unified concept for defining conservation units. Molecular Ecology.

[EYU023C20] GITTENBERGER E., BACKHUYS W., RIPKEN T.E.J. (1970). De Landslakken van Nederland.

[EYU023C21] GITTENBERGER E., KOKSHOORN B., Morris S., Vosloo A. (2008). Evolutionary inequality: comparing phylogenetic relationships with other biological properties. Molecules to migration: the pressures of life. 4th CPB meeting in Africa: MARA 2008.

[EYU023C22] GITTENBERGER E., NEUTEBOOM W.H. (1991). On *Trichia alpicola* (Eder, 1921) from Switzerland (Mollusca: Gastropoda Pulmonata: Hygromiidae) and the spiral structure on its shell. Zoologische Mededelingen.

[EYU023C23] GIUSTI F., MANGANELLI G. (1987). On some Hygromiidae (Gastropoda: Helicoidea) living in Sardinia and in Corsica (Studies on the Sardinian and Corsican malacofauna VI). Bolletino Malacologico.

[EYU023C24] HADLEY A. (2010). http://www.http://www.hadleyweb.pwp.blueyonder.co.uk.

[EYU023C25] HORSÁK M., HÁJEK M., TICHÝ L., JUŘÍČKOVÁ L. (2007). Plant indicator values as a tool for land mollusc autecology assessment. Acta Oecologica.

[EYU023C26] HORSÁK M., ŠKODOVÁ J., MYŠÁK J., ČEJKA T., LOŽEK V., HLAVÁČ J.Č. (2010). *Pupilla pratensis* (Gastropoda: Pupillidae) in the Czech Republic and Slovakia and its distinction from *P. muscorum* and *P. alpicola* based on multidimensional analysis of shell measurements. Biologia.

[EYU023C27] HOTOPP K.P., PEARCE T.A., NEKOLA J.C., SCHMIDT K. (2010). New land snail (Gastropoda: Pulmonata) distribution records for New York State. Proceedings of the Academy of Natural Sciences of Philadelphia.

[EYU023C28] HRABÁKOVÁ M., JUŘIČKOVÁ L., PETRUSEK A. (2006). Problematika rodu *Trochulus* (Mollusca, Gastropoda) v České republice. Sborník abstraktů Zoologické dny Brno.

[EYU023C29] JORDAENS K., VAN DONGEN S., VAN RIEL P., GEENEN S., VERHAGEN R., BACKELJAU T. (2002). Multivariate morphometrics of soft body parts in terrestrial slugs: comparison between two datasets, error assessment and taxonomic implications. Biological Journal of the Linnean Society.

[EYU023C30] JUŘIČKOVÁ L., LOŽEK V. (2008). Molluscs of the Krkonoše Mts (Czech Republic). Malacologica Bohemoslovaca.

[EYU023C31] KERNEY M.P., CAMERON R.A.D., JUNGBLUTH J.H. (1983). Die Landschnecken Nord- und Mitteleuropas.

[EYU023C32] KLEMM W. (1974). Die Verbreitung der rezenten Land-Gehäuse-Schnecken in Österreich. Denkschriften der Österrerreichischen Akademie der Wissenschaften (mathematisch-naturwissenschaftliche Klasse).

[EYU023C33] KLÖTI-HAUSER E. (1920). Beiträge zur Anatomie des Geschlechtsapparates einiger schweizerischer *Trichia- (Fruticicola- Helix-)* Arten.

[EYU023C34] KRUCKENHAUSER L., DUDA M., BARTEL D., SATTMANN H., HARL J., KIRCHNER S., HARING L. (2014). Paraphyly and budding speciation in hairy snails (Gastropoda, Pulmonata, Hygromiidae). Zoologica Scripta.

[EYU023C35] KRUCKENHAUSER L., HARL J., SATTMANN H. (2011). Optimized drowning procedures of pulmonate landsnails allowing subsequent DNA analysis and anatomical dissections. Annalen des Naturhistorischen Museums.

[EYU023C36] LOŽEK V. (1956). Klíč československých měkkýšů.

[EYU023C37] LOŽEK V. (1963). Quartärmollusken der Tschechoslowakei. Rozpravý ústředního ústavu geologického.

[EYU023C38] NAGGS F. (1985). Some preliminary results of a morphometric multivariate analysis of the *Trichia* (Pulmonata: Helicidae) species group in Britain. Journal of Natural History.

[EYU023C39] PAWŁOWSKA-BANASIAK E. (2008). Shell, genital and colour variation in *Perforatella incarnata* (O.F. Müller, 1774) and *P. vicina* (Rossmässler, 1842) (Gastropoda: Pulmonata: Helicidae). Folia Malacologica.

[EYU023C40] PFENNINGER M., HRABÁKOVÁ M., STEINKE D., DÉPRAZ A. (2005). Why do snails have hairs? A Bayesian inference of character evolution. BioMedCentral Evolutionary Biology.

[EYU023C41] PINCEEL J., JORDAENS K., VAN HOUTTE N., DE WINTER A.J., BACKELJAU T. (2004). Molecular and morphological data reveal cryptic taxonomic diversity in the terrestrial slug complex *Arion subfuscus/fuscus* (Mollusca, Pulmonata, Arionidae) in continental north-west Europe. Biological Journal of the Linnean Society.

[EYU023C42] PROĆKÓW M. (2009). The genus *Trochulus* Chemnitz, 1786 (Gastropoda: Pulmonata: Hygromiidae) – a taxonomic revision. Folia Malacologica.

[EYU023C43] PROĆKÓW M., MACKIEWICZ P., PIEŃKOWSKA J.R. (2013). Genetic and morphological studies of species status for poorly known endemic *Trochulus phorochaetius* (Bourguignat, 1864) (Gastropoda: Pulmonata: Hygromiidae), and its comparison with closely related taxa. Zoological Journal of the Linnean Society.

[EYU023C44] R DEVELOPMENT CORE TEAM (2012). R: a language and environment for statistical computing.

[EYU023C45] REISCHÜTZ A., REISCHÜTZ P.L., Zulka P. (2007). Rote Liste der Weichtiere (Mollusca) Österreichs. Rote Listen gefährdeter Tiere Österreichs. Checklisten, Gefährdungsanalysen, Handlungsbedarf.

[EYU023C46] REISCHÜTZ P.L. (1999). Bestimmung und Sektion von Nacktschnecken. Heldia, Sonderheft.

[EYU023C47] SAUER J., HAUSDORF B. (2012). A comparison of DNA-based methods for delimiting species in a Cretan land snail radiation reveals shortcomings of exclusively molecular taxonomy. Cladistics.

[EYU023C48] SCHILEYKO A.A. (1978). On the systematics of *Trichia* s. lat. (Pulmonata: Helicoidea: Hygromiidae). Malacologia.

[EYU023C49] SCHILEYKO A.A. (2006). Treatise on recent terrestrial pulmonate molluscs. Part 14: Helicodontidae, Ciliellidae, Hygromiidae. Ruthenica, Supplement.

[EYU023C50] TABACHNIK B., FIDELL L. (1996). Using multivariate statistics.

[EYU023C51] TURNER H., KUIPER J.G.J., THEW N., BERNASCONI R., RÜETSCHI J., WÜTHRICH M., GOSTELI M. (1998). Fauna Helvetica 2. Atlas der Mollusken der Schweiz und Lichtensteins.

[EYU023C52] VAN HUSEN D. (1997). Die Ostalpen in den Eiszeiten..

[EYU023C53] WAGNER A.J. (1915). Beiträge zur Anatomie und Systematik der Stylommatophoren aus dem Gebiet der Monarchie und der angrenzenden Balkanländer. Denkschriften der Österreichischen Akademie der Wissenschaften (mathematisch-naturwissenschaftliche Klasse).

